# The developing tendon and enthesis are hypoxic and rely on hypoxia-inducible factor 1a during postnatal development

**DOI:** 10.1242/dev.205458

**Published:** 2026-06-22

**Authors:** Stephanie S. Steltzer, Nicole Migotsky, Tessa Phillips, Syeda N. Lamia, Bachir A. Abeid, Kiwon Lee, Seung-Ho Bae, Connor Leek, Sydney Grossman, Moaid Shaik, Allison Risha, Kaitlyn Frey, Claudia Loebel, Jun Hee Lee, Yatrik M. Shah, Adam C. Abraham, Megan L. Killian

**Affiliations:** ^1^ Department of Orthopedic Surgery, Michigan Medicine, Ann Arbor, MI 48109, USA; ^2^ Department of Molecular and Integrative Physiology, Michigan Medicine, Ann Arbor, MI 48109, USA; ^3^ Department of Bioengineering, University of Pennsylvania, Philadelphia, PA 19104, USA; ^4^ Department of Internal Medicine, Division of Gastroenterology, Michigan Medicine, Ann Arbor, MI 48109, USA

**Keywords:** Tendon, Enthesis, Hypoxia, HIF-1α, Mouse

## Abstract

The enthesis is a specialized tissue crucial for transmission of muscle force from tendon to bone, yet its postnatal development is not fully understood. In this study, we investigated if and how hypoxia inducible factor 1 alpha (HIF-1α) regulates enthesis cell adaptation, e.g. cell survival and extracellular matrix (ECM) deposition. We investigated the spatial and temporal dynamics of hypoxia in the murine Achilles tendon enthesis and found that, whereas neonatal tendons rapidly resolve hypoxia after birth, the enthesis maintains a hypoxic niche until postnatal day 5, mirroring a hypoxic gradient observed in growth plates. Genetic disruption of *Hif1a* in mouse tendon/enthesis-resident cells (cKO) resulted in pronounced deficits in grip strength, abnormal tendon–bone attachment morphology, disrupted calcaneal architecture, impaired mineralization and significant ECM disorganization. We found persistent cell death and loss of collagen organization in cKO entheses. *In vitro*, *Hif1a*-deficient tendon fibroblasts exhibited blunted transcriptional responses to hypoxia, altered metabolic gene expression and disrupted ECM deposition. Collectively, our findings illuminate hypoxia as a sustained niche in the postnatal enthesis, with HIF-1α required for cell survival, ECM organization and structural integrity of entheses.

## Teaser

**Summary:** A sustained hypoxic niche in the postnatal mouse enthesis requires HIF-1α to preserve cell survival and extracellular matrix gradients, and losing HIF-1α disrupts the structure and function of the tendon–bone attachment.

## INTRODUCTION

The tendon–bone attachment (i.e. enthesis) is essential for the transmission of muscle forces to bone (Benjamin et al., 2006). The progenitor pool that forms the enthesis also contributes to tendon development and is identified by the transcription factor gene scleraxis (*Scx*) (Blitz et al., 2013; Kult et al., 2021; Schweitzer et al., 2001). The enthesis develops analogously to the skeletal growth plate of long bones in a process whereby cartilage is replaced with bone via endochondral ossification (Wang et al., 2013; Schwartz et al., 2015; Rossetti et al., 2017). However, while the growth plates eventually fuse, the fibrocartilage enthesis remains static throughout the lifespan and has been likened to an ‘arrested’ growth plate (Schwartz et al., 2012).

During embryonic development, the growth plates of long bones maintain a hypoxic niche that resolves with vascularization (Benjamin et al., 2002; Schipani et al., 2001; Zelzer et al., 2004). Hypoxia inducible factor 1 alpha (HIF-1α) is a master transcriptional regulator of cell survival in hypoxic environments and modulates metabolic pathways that promote the deposition of extracellular matrix (ECM) and vascularization (Amarilio et al., 2007; Bentovim et al., 2013). HIF-1α is required for the survival of growth plate chondrocytes during development, and loss of *Hif1a* results in cell death and structural abnormalities in developing bone (Schipani et al., 2001; Bentovim et al., 2013). However, the enthesis is well known to maintain limited vascularization, suggesting that cellular hypoxia may play a sustained role in its maintenance.

Hypoxia also regulates cell proliferation, and tendon fibroblasts (TFs) are highly proliferative during embryonic tendon development prior to when high amounts of ECM are deposited. However, the cells in the mature tendon do not retain the same level of proliferation (Grinstein et al., 2019). Likewise, the density of resident cells in the postnatal enthesis decreases as its resident cells secrete and remodel their ECM, with little to no proliferation during this time (Ganji et al., 2023). The enthesis consists of both cellular and ECM gradients from tendon to bone that are established during postnatal growth (Kult et al., 2021; Schwartz et al., 2012; Dyment et al., 2015). The gradient-like distribution of cells in the enthesis as well as tendon may be driven by changes in the oxygen environment, yet this spatial distribution of hypoxia has been unclear until now.

In this study, we hypothesized that hypoxia is sustained in postnatal enthesis and that enthesis-resident cells rely on HIF-1α for their survival and ECM deposition. We first explored when enthesis-resident cells experience hypoxic stress during embryonic and neonatal growth in mice. We then identified a crucial role of *Hif1a* for enthesis cell survival, ECM organization, spatial transcriptome, and biomechanical function in the mouse Achilles tendon and enthesis with targeted deletion of HIF-1α in the *Scx* lineage *in vivo*. Because the enthesis is composed primarily of Scx-lineage fibroblasts, we further investigated if and how murine fibroblasts respond *in vitro* to low oxygen environments. To do this, we measured the transcriptional response as well as cell and ECM dynamics of fibroblasts in hypoxia or following loss of *Hif1a in vitro*.

## RESULTS

### Enthesis, but not tendon, remains hypoxic during early postnatal development

**Fig. 1.**
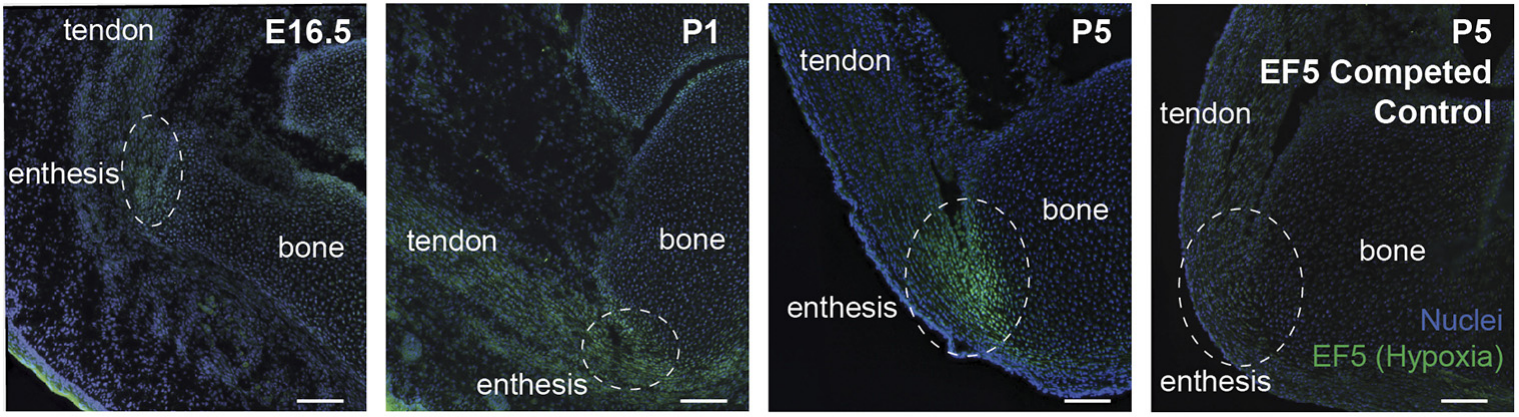
**Enthesis, but not tendon, remains hypoxic during postnatal growth.** Accumulation of EF5 (hypoxia marker, green fluorescence) becomes more localized to the C57BL6/J mouse Achilles enthesis during early postnatal development (E16.5) up to P1 and P5 compared to tendon and bone. EF5, green fluorescence; nuclei, blue fluorescence. Right-hand panel is a competed control. *n*≥3 C57BL6J embryos or mice per time point. Enthesis is encircled by dashed line. Scale bars: 200 μm.

Given the intrinsic link between hypoxia and HIF-1α, we first determined whether a hypoxic environment exists at the embryonic and neonatal Achilles tendon–bone enthesis using 2-(2-nitro-1*H*-imidazol-1-yl)-*N*-(2,2,3,3,3-pentafluoro-propyl) acetamide (EF5) staining, which forms intracellular adducts in hypoxic conditions (<10% O_2_) that can be visualized via immunostaining. EF5 staining for hypoxic cells revealed that both embryonic tendon and enthesis experience hypoxia, with strong EF5 staining at embryonic day (E) 16.5 and postnatal day (P) 1 (Fig. 1). Additionally, the enthesis remained hypoxic until P5, whereas the developing tendon was no longer experiencing hypoxic stress at P5 (Fig. 1).

### Loss of *Hif1a* in tendon progenitors leads to impaired grip force and deficient tendon/enthesis function

**Fig. 2.**
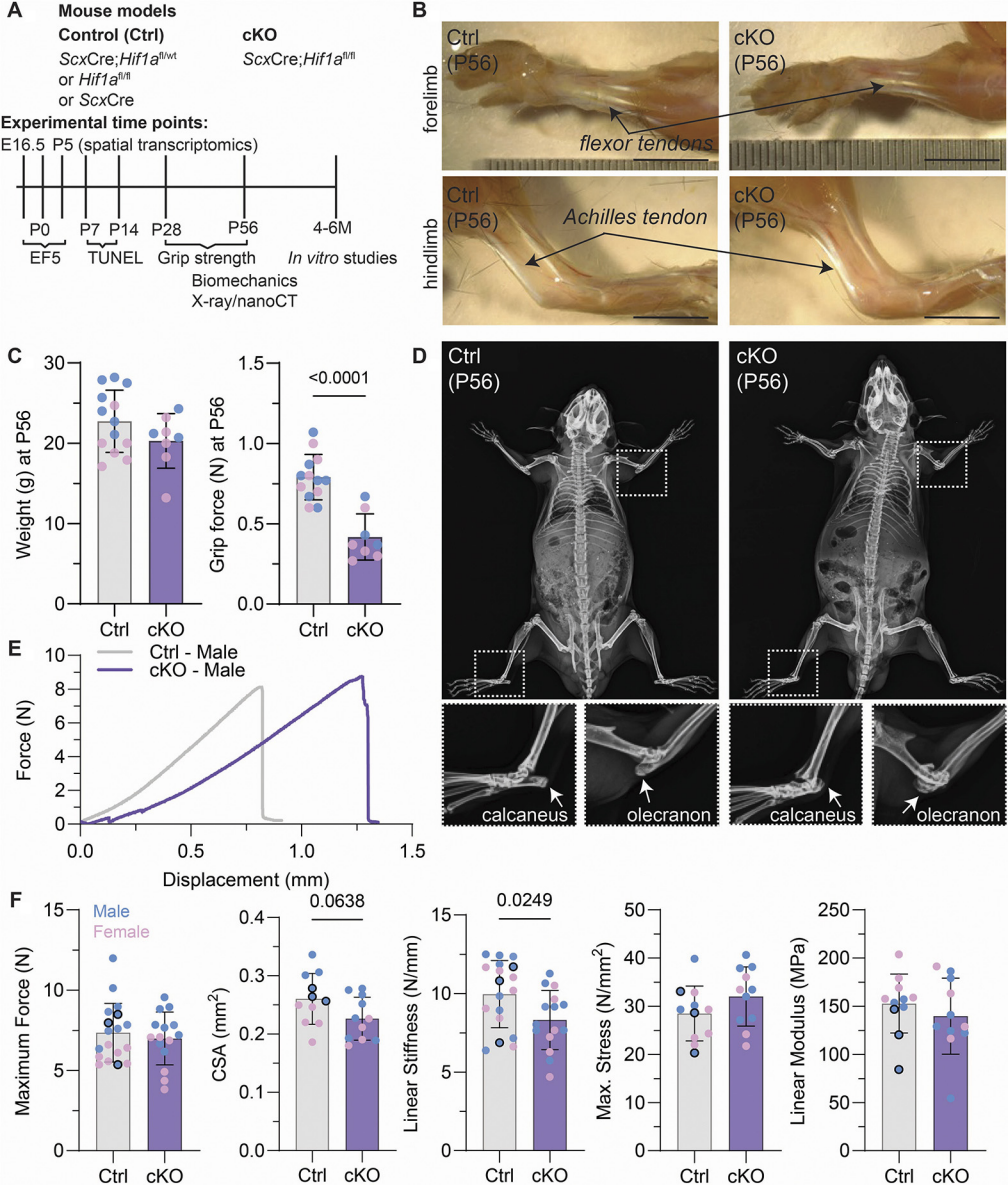
**Loss of *Hif1a* in tendon leads to impaired grip force and deficient tendon function.** (A) Mouse genotypes used (Ctrl and cKO) and experimental time points to evaluate the effects of Hif1a loss in Scx-lineage cells (*Hif1a^flox/flox^*;*ScxCre*; cKO). E, embryonic day; P, postnatal day; M, months. (B) Force-generating tendons pattern normally in mice lacking *Hif1a* in tendons. Morphology of the forelimb (e.g. flexor tendons) and hindlimb (e.g. Achilles tendon) tendons in cKO mice were unremarkable compared to control (Ctrl) mice at P56. Scale bars: 0.5 mm. (C) Knockdown of *Hif1a* did not affect mouse weight at P56. Both male (blue) and female (pink) cKO mice had reduced forepaw grip strength. *n*=15 for Ctrl and *n*=11 for cKO at 4 weeks of age; *n*=13 for Ctrl and *n*=8 for cKO at 8 weeks of age; two-way ANOVA (sex/genotype) with Dunnett's corrected multiple comparisons. (D) Planar X-rays of cKO mice at P56 show marked disruptions in tendon–bone attachment sites compared to control, most notably in the elbow (olecranon; white dotted box) and ankle (calcaneus; white dotted box) (both shown at higher magnification beneath). (E) Representative force versus displacement curve for P56 Ctrl and cKO Achilles tendon-enthesis. (F) Ultimate load (N) of Achilles tendon-entheses at P56 was similar in cKO mice compared to control. Cross-sectional area (CSA; mm) of Achilles tendons was reduced in cKO mice compared to control. Achilles tendon-enthesis stiffness (N/mm) was decreased in both male and female cKO mice compared to control, while maximum stress and linear modulus remained similar across genotypes. Cre-negative, *n*= 15; Cre-positive, *n*=3 males; cKO mice, *n*=15. One-way ANOVA with Dunnett's corrected multiple comparisons. Points outlined in black indicate *Scx*Cre-positive control animals. (G) Representative X-ray images of Achilles tendons and calcanei in boots after biomechanics testing revealed unique failure modes in cKO tendons (primarily in the enthesis or midsubstance) compared to Ctrl tendons (primarily in the growth plate). Data are presented as mean±s.d. *P*-values are shown on graphs.

**Fig. 3.**
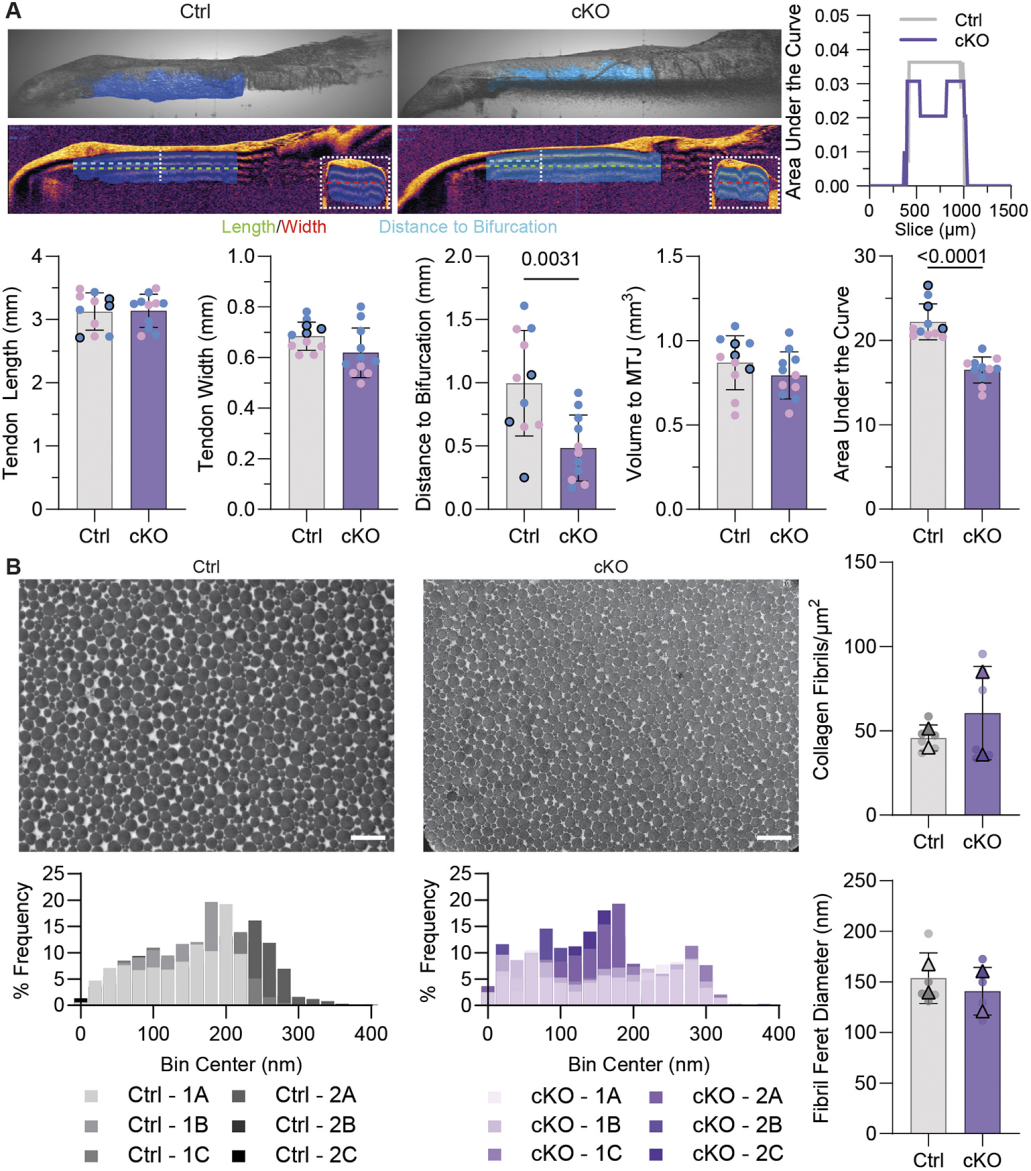
**Loss of *Hif1a* leads to impaired organization of the mouse Achilles tendon compared to Ctrl.** (A) Representative images of Achilles tendon masks generated in Dragonfly ORS using retardation and intensity images. Achilles tendon length, width and distance to bifurcation were quantified using OCT. Blue indicates mask of tendon; the green dashed lines represent tendon length; red dashed lines represent tendon width; the white dashed line in the longitudinal plane indicates where the bifurcation starts; the cross-sectional view of each tendon at the bifurcation is highlighted in the subset box with a white dashed outline. FFT analyses of the frequency of retardation show that tendons lacking *Hif1a* have reduced collagen organization (smaller AUC) with focal disorganization in the tendon core that does not exist in age-matched Ctrl tendons. The same animals used for biomechanical testing were used for OCT analyses. Blue circles, male; pink circles, female; outlined circles, ScxCre-only (no floxed alleles). MTJ, muscle-tendon junction. (B) TEM images from cross-sections of Ctrl and cKO Achilles tendon revealed a broader distribution of collagen fibril size in cKO tendons compared to Ctrl. Three consecutive sections (*n*=3; circles) from two animals each (*N*=2; triangles). Data are presented as mean±s.d. *P*-values are shown on graphs (two-tailed unpaired *t*-test). Scale bars: 600 nm.

**Fig. 4.**
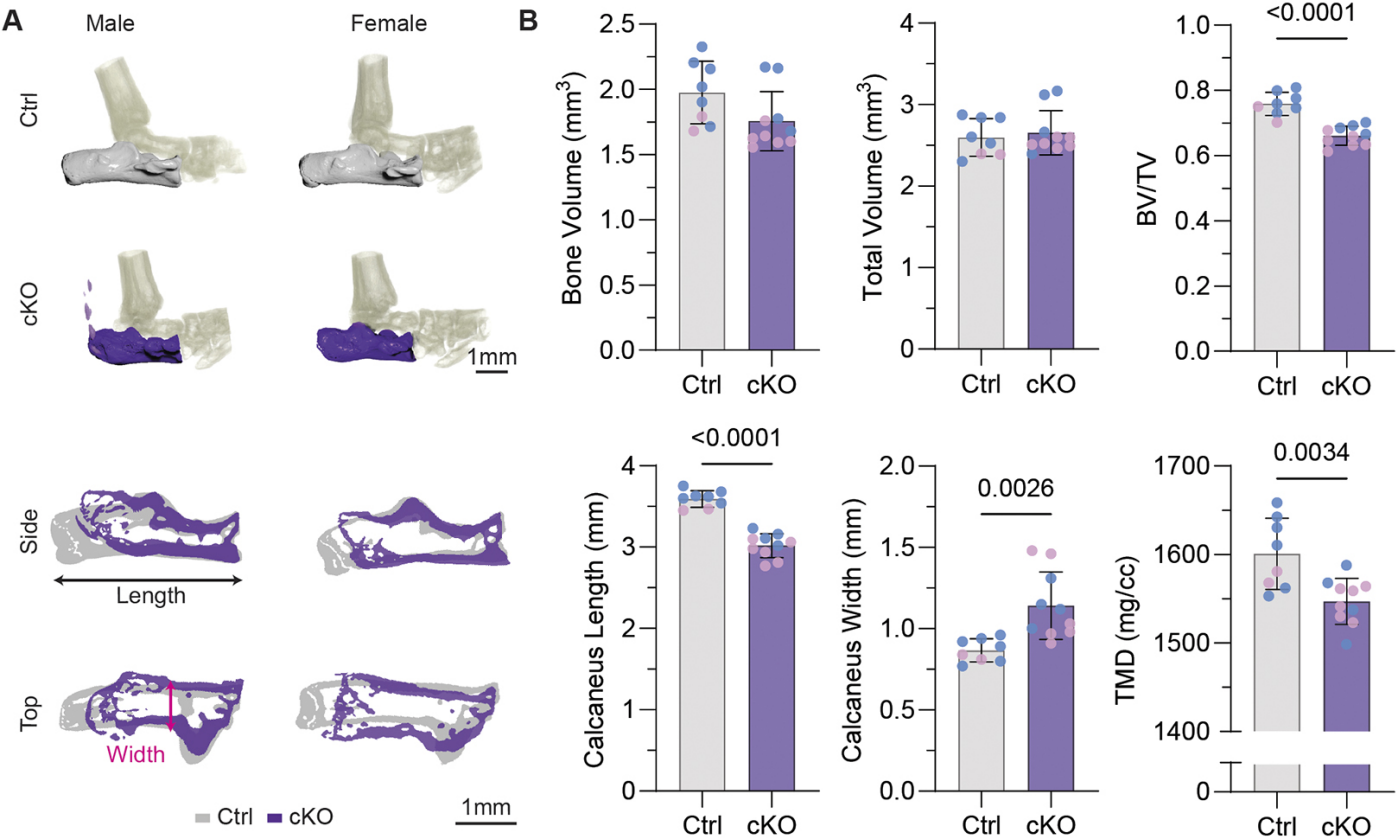
**Loss of *Hif1a* disrupts calcanei shape and size at the Achilles tendon insertion site.** (A) 3D and 2D nanoCT reconstructions of both male and female P56 cKO calcanei revealed that HIF-1α is required to maintain proper calcaneus shape and size near the Achilles tendon insertion site compared to the control. Scale bars: 1 mm. (B) NanoCT analyses of the calcaneus revealed a decrease in bone volume fraction (BV/TV) and calcaneus length of P56 cKO mice compared to control with an increase in calcaneus width in cKO mice compared to control. Tissue mineral density (TMD) also decreased in cKO mice compared to control. Mineralization was evident in the male cKO Achilles tendons. Ctrl (Cre-negative), *n*=6 males and *n*=2 females; cKO, *n*=4 males and *n*=6 females; male and female mice annotated as blue and pink dots, respectively. Data are presented as mean±s.d. *P*-values are shown on graphs (one-way ANOVA with Dunnett's corrected multiple comparisons).

We investigated the Achilles tendon and enthesis of mice with or without *Hif1a* at several time points throughout development (Fig. 2A). In mice lacking *Hif1a* in *Scleraxis*-Cre (*Scx*Cre) cells (*Hif1a^ScxCre^*; cKO), overall tendon patterning appeared grossly normal; however, tendon fusion was disrupted compared to 8-week (P56) littermate controls (Ctrl), which was visualized using optical coherence tomography (OCT) with polarization (Figs 2B and 3). We found that cKO Achilles tendons had less overall birefringence, indicative of collagen organization, compared to Ctrl [smaller area under the curve (AUC); Fig. 3A; measured by 2D Fast Fourier Transform (FFT) analyses of OCT image slices]. Additionally, cKO tendons had a bifurcating region of increased disorganization in its central core (detected visually using OCT and algorithmically using FFT), which was not present in Ctrl tendons (Fig. 3A). Furthermore, forelimb grip strength was significantly lower for cKO mice compared to Ctrl, regardless of sex (Fig. 2C), but Ctrl and cKO mice were comparable in weight (Fig. S1). The skeletal morphology, visualized using planar X-ray, showed that joint congruency in cKO mice were severely impaired, especially at sites where tendons attach to bone (e.g. the calcaneus of the heel and olecranon of the elbow; Fig. 2D). Additionally, the Achilles tendon length, width and volume were comparable between adult Ctrl and cKO mice. However, we visualized using OCT that cKO Achilles tendons failed to fuse and remained bifurcated distally compared to Ctrl tendons by 8 weeks of age (Fig. 3A) with an increased collagen fibril size distribution as seen by transmission electron microscopy (TEM) (Fig. 3B). We evaluated calcaneal morphometry using nano-computed tomography (nanoCT) and found that cKO calcanei had incomplete and irregular bone formation adjacent to the Achilles enthesis in both male and female mice (Fig. 4A). While Ctrl mice did not present with any mineralization in their tendons by 8 weeks of age, we observed mineral accumulation in male, but not female, cKO mice at the distal Achilles tendon [Fig. 4A; 0.024±0.013 mm^3^ (mean±s.d.)]. Calcaneal bone volume ratio (bone volume/total volume of the entire calcaneus including marrow space) was also reduced in cKO mice compared to Ctrl (Fig. 4B). Additionally, cKO calcanei exhibited notable morphological disruptions and were both shorter and wider than Ctrl calcanei (Fig. 4A,B). The widening of the calcanei in cKO mice was especially pronounced near the calcaneal tuberosity (Fig. S2) and accounted for 51.1% of the shape variation compared to Ctrl calcanei. Surprisingly, these major differences in bone and tendon structure did not translate to significant differences in biomechanical strength of the Achilles tendons, as adult Ctrl and cKO tendons did not show differences in maximum (failure) force or stress (Fig. 2E,F). However, linear stiffness was significantly lower in cKO compared to Ctrl tendons, and the cross-sectional area (CSA) of the cKO tendons was also smaller (*P*=0.0638) compared to Ctrl tendons (Fig. 2F), and this did not translate to differences between groups in CSA-normalized stiffness (i.e. linear modulus; Fig. 2F). Despite this, the failure modes of tendons during biomechanical testing were different between Ctrl and cKO groups, in which nearly all Ctrl Achilles tendons failed at the growth plate and all but one cKO tendon failed at either the Achilles enthesis or tendon midsubstance (Fig. 2G). To note, in using this setup for uniaxial tensile testing of the Achilles tendon, failure at the immature growth plate in 8-week-old wild-type calcanei were visualized and is a limitation in this study as the failure is not exclusively within the tendon midsubstance. The difference in failure modes was possibly due to poor bone quality at the enthesis in cKO mice and preservation of the calcaneal growth plate in the Ctrl but not cKO heels. The significant differences in tendon bifurcation, visualized using OCT, as well as differences in failure models, led us to further explore the role of HIF-1α in tendon and enthesis organization during key developmental stages, when new ECM is being deposited and remodeled (Dyment et al., 2015; Galatz et al., 2007).

### Loss of *Hif1a* disrupts ECM organization and cell viability in the postnatal enthesis

**Fig. 5.**
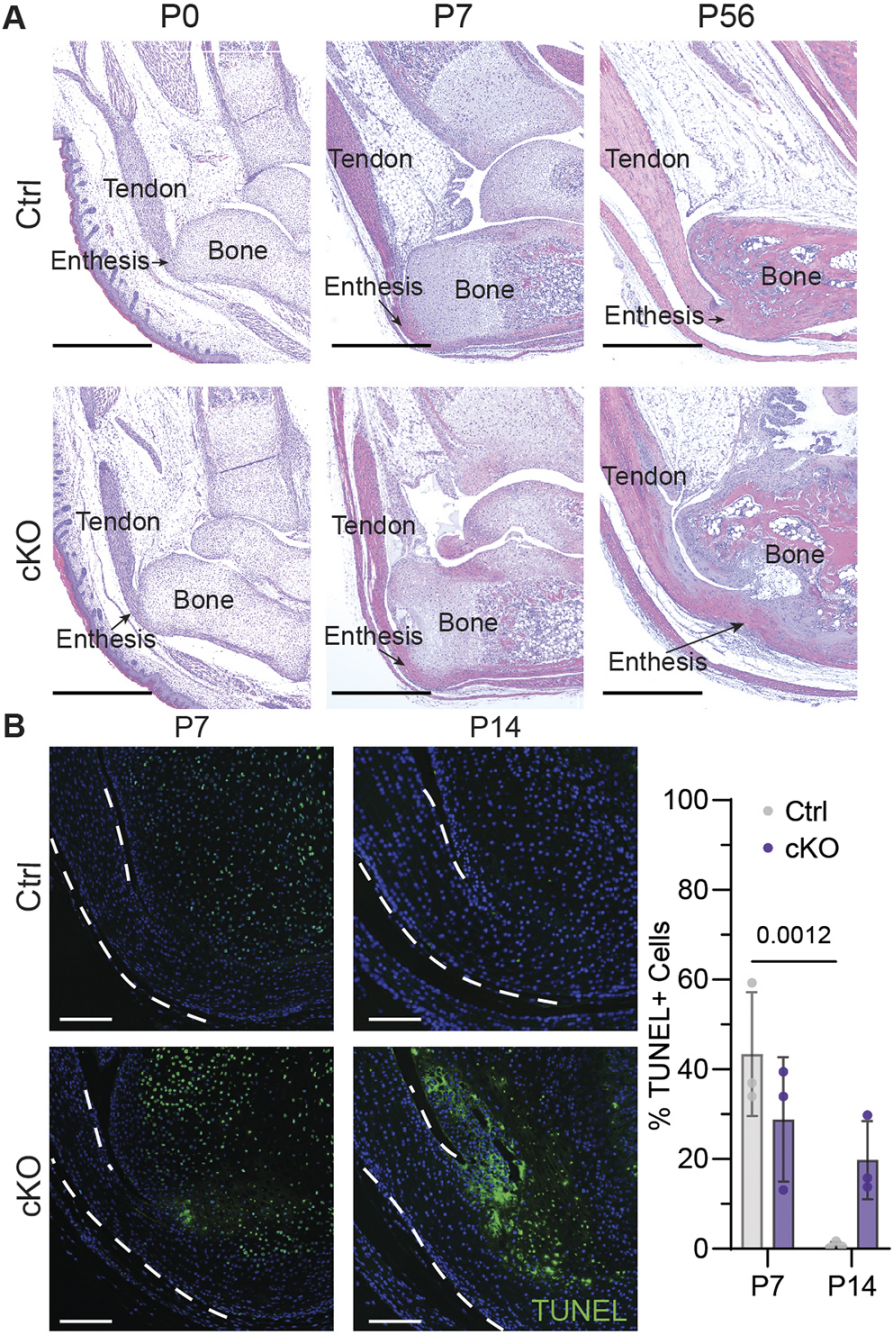
**The developing tendon and enthesis are reliant on *Hif1a* for structure and cell survival.** (A) Hematoxylin and Eosin staining at P0 showed unremarkable differences due to *Hif1a* knockout, whereas disorganized cells and ECM are present at P7 and P56 entheses. Ctrl (Cre-negative), *n*=3 per time point; cKO, *n*=3 per time point. Scale bars: 1 mm. (B) Cells in the insertional tendon (white dashed line) and enthesis exhibit more DNA damage and cell death (TUNEL^+^ cells, green fluorescence) in P14 cKO mice compared to control. Scale bars: 200 µm. Ctrl (Cre-negative), *n*=3 P7 and *n*=3 P14; cKO, *n*=3 P7 and *n*=3 P14. Data are presented as mean±s.d. *P*-values are shown on graphs (two-way ANOVA with Tukey's multiple comparisons test).

Normal fibrocartilage development occurs postnatally in the mouse enthesis, with a postnatal mineralization and transition between mineralized and unmineralized fibrocartilage established around 2 weeks of age (∼P14) (Schwartz et al., 2012). In the normally developing Achilles enthesis, we observed the cellular gradient by P7 and the transitional ECM between tendon and cartilage template by P14 (Fig. 5A). The fully mature Achilles enthesis was established by P56 (Fig. 5A). In the cKO enthesis, the tendon-bone enthesis was established at P0; however, by P14 and up to P56, the Achilles tendon and enthesis exhibited more cell and ECM disorganization (Fig. 5A, Fig. S3A). The deformity in the mature enthesis was characterized by focal bone loss proximal to the attachment site, focal regions of hypo- and hypercellularity in the insertional tendon, and ECM disorganization at the tendon–bone interface (Fig. 5A, Fig. S3A). We also found that cell death (detected by TUNEL staining) was consistent between genotypes at P7, remained elevated in cKO but not Ctrl entheses by P14 (Fig. 5B) and resolved to comparable levels by P56 (Fig. S3B). This suggests that *Hif1a* plays a crucial role in enthesis cell survival during postnatal maturation of the fibrocartilage enthesis.

### *Hif1a* is required for the formation of a structural gradient at the Achilles enthesis

**Fig. 6.**
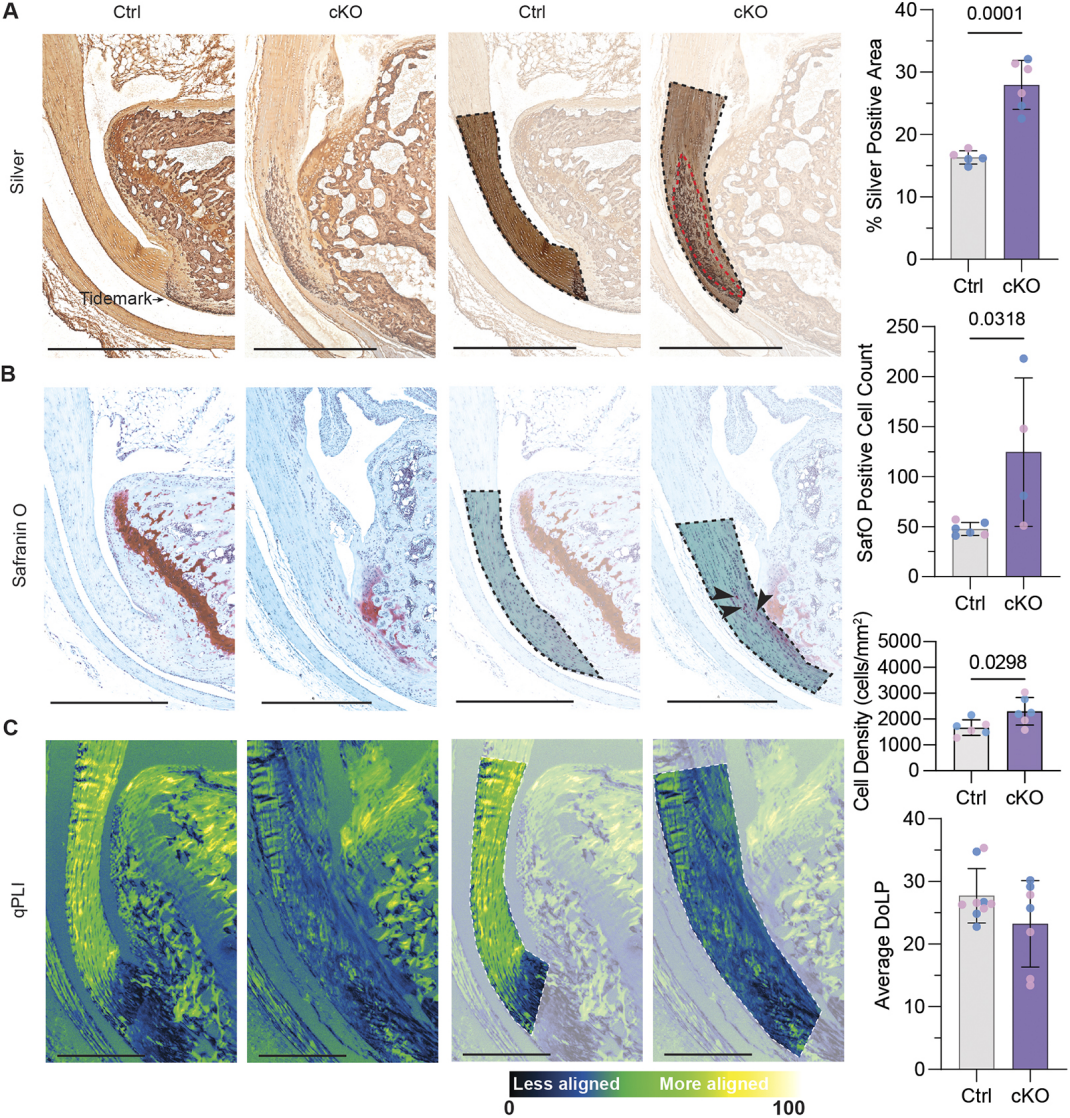
***Hif1a* is required for structural integration of the tendon into the bone.** (A) Silver nitrate sections of cKO mouse Achilles enthesis contained a build-up of DNA and protein compared to the age-matched Cre-control (Ctrl) at P56. Increased areas of protein accumulation (red dashed line) occurred in cKO mouse entheses with poorly defined secondary ossification center compared to Ctrl. Ctrl (Cre-negative), *n*=3 male and *n*=2 female; cKO, *n*=3 male and *n*=3 female. (B) Safranin O sections of P56 cKO mouse Achilles enthesis revealed cKO growth plates were poorly organized and an increased number of proteoglycan-rich cells (black arrowheads) were apparent in the cKO insertional tendon when compared to Ctrl. Ctrl (Cre-negative), *n*=4 male and *n*=2 female; cKO, *n*=2 male and *n*=2 female. Total cell density within the insertional tendons was significantly higher for cKO tendons compared to Ctrl. Ctrl (Cre-negative), *n*=3 male and *n*=3 female; cKO, *n*=3 male and *n*=3 female. (C) Degree of linear polarization images of Achilles insertional tendon identified decreased collagen organization in P56 cKO mice compared to Ctrl. Ctrl (Cre-negative), *n*=4 male and *n*=5 female; cKO, *n*=3 male and *n*=4 female. Data are presented as mean±s.d. *P*-values are shown on graphs (one-way ANOVA with Dunnett's corrected multiple comparisons). DoLP, degree of linear polarization; qPLI, quantitative polarized light imaging. Black dashed outlines in A and B highlight the regions used for quantification of Silver-positive and SafO-positive areas; the white dashed outlines in C highlight the regions used for qPLI. Scale bars: 1 mm.

Using silver nitrate staining, which is commonly used to stain osteocytes in bone (Dole et al., 2021), we observed a well-defined tidemark that separates mineralized and unmineralized fibrocartilage in the Ctrl Achilles enthesis by P56 (Fig. 6A). However, in age-matched mice, cKO entheses had diffuse silver nitrate staining in the insertional tendons that was not present in Ctrl tendons (Fig. 6A,B). Additionally, the distinct tidemark was not present in cKO entheses (Fig. 6A). We also observed, using Safranin O staining for acidic proteoglycans and glycosaminoglycans (commonly used to stain cartilage), that Ctrl insertional tendons were sparse in proteoglycan-rich cells while cKO tendons had significantly more proteoglycan-rich cells adjacent to the articular surface (Fig. 6B). Additionally, cKO insertional tendons had more cells (higher cell density) compared to Ctrl tendons at P56 (Fig. 6B). This may be caused by a localized shift in tendon compression in cKO mice (Rossetti et al., 2017; Couchman and Pataki, 2012; Ansorge et al., 2011). We also saw a severe disruption and loss of Safranin-O^+^ growth plates of cKO mice, which remained distinct in Ctrl calcanei at P56 (Fig. 6B). To further interrogate ECM organization, we used Picrosirius Red staining and quantitative polarized light imaging to quantify collagen alignment in the insertional tendon and found that Ctrl tendons had highly aligned collagen at P56 (Fig. 6C). However, cKO tendons were less uniformly aligned, as quantified by a reduced average degree of linear polarization (DoLP). Taken together, these data suggest that survival of cells within and adjacent to the enthesis is necessary to generate the gradient ECM needed for proper enthesis maintenance.

### Knockdown of *Hif1a* in mouse tail TFs desensitizes these cells to hypoxic conditions compared to normoxic conditions

**Fig. 7.**
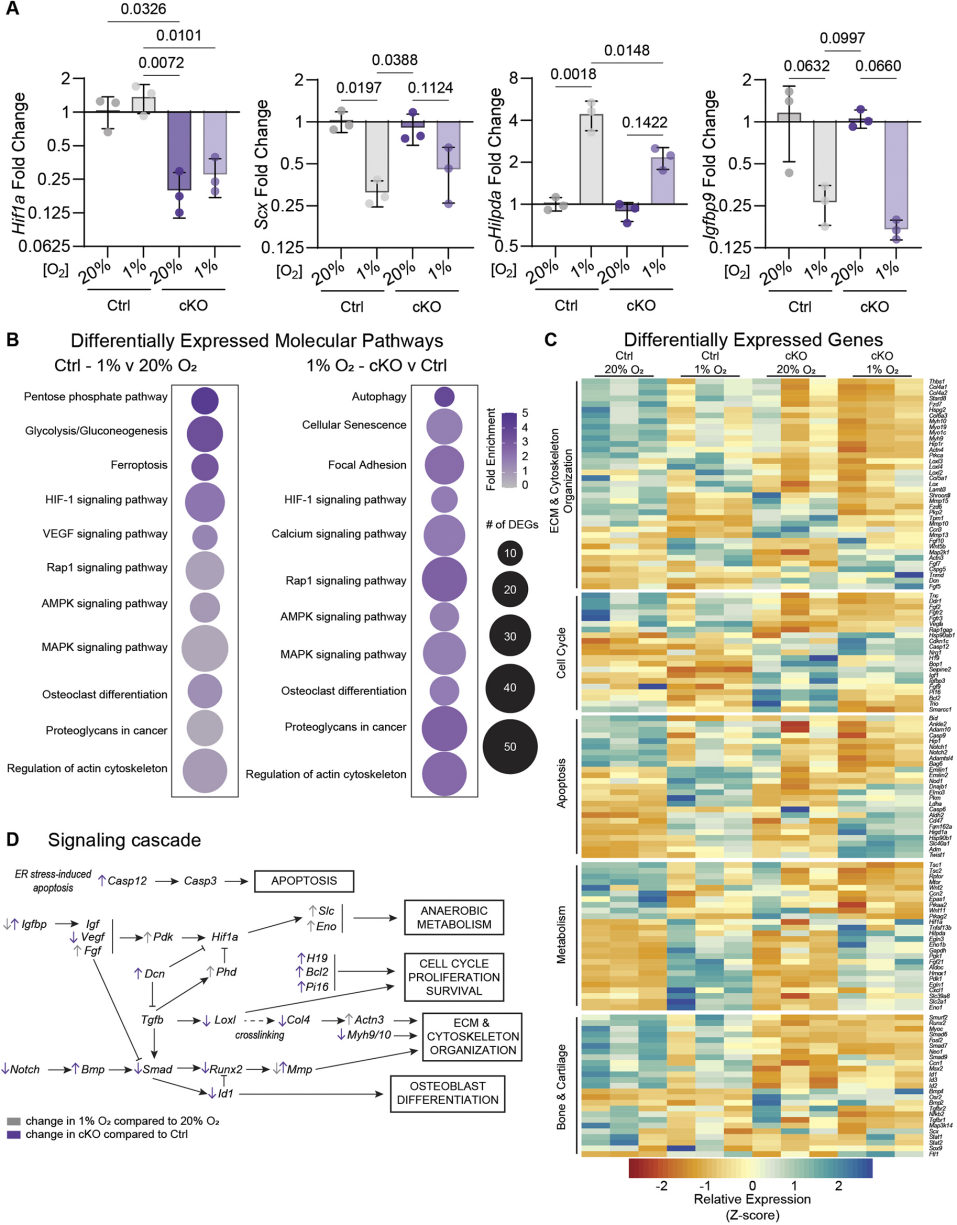
**Knockdown of *Hif1a* in mouse tail TFs desensitizes TFs to hypoxic conditions (1% O_2_) compared to normoxic conditions (20% O_2_).** (A) *Hif1a* relative expression is reduced in mouse tail TFs cultured from mice lacking *Hif1a*. TFs cultured for 1 week in hypoxia express reduced tenogenic (*Scx*) and as well as activated hypoxia-related (*Hilpda*) genes. Each dot represents a biological replicate (*n*=3 per condition); (2^−ΔCq^; *Actb*, *Polr2a* reference genes). Bars show mean±s.d. of ΔC_q_; two-way ANOVA tests with Dunnett's multiple comparisons for hypoxia and genotype. (B) Quantification of KEGG pathways found during bulk RNA-seq in Ctrl 1% O_2_ compared to Ctrl 20% O_2_ and cKO 1% O_2_ compared to Ctrl 1% O_2_. (C) Heatmap depicting DEGs affiliated with ECM and cytoskeleton organization, cell cycle, apoptosis, metabolism, and bone and cartilage. Differentially expressed genes were determined using a fold change cutoff of 1.5 and a *P*-value cutoff of 0.05. (D) Signaling diagram created from differentially expressed genes (*n*=3 per condition).

Given the established hypoxic gradient at the enthesis and the clear disruption in tendon and enthesis organization in cKO mice, we then investigated the role of HIF-1α in regulating oxygen-dependent transcriptional programs and ECM in TFs. We found that mouse tail TFs from *Hif1a* cKO mice had reduced sensitivity to hypoxia (1% O_2_) after 1 week in culture, as indicated by damped expression of a known hypoxia-sensitive gene hypoxia inducible lipid droplet associated [*Hilpda* (also known as *HIG-2*); Fig. 7A]. In Ctrl TFs, we found that 1% O_2_ led to downregulation of the tenogenic transcription factor *Scx*. A known gene target of *Hif1a*, Insulin-like growth factor binding protein 9 (*Igfbp9*; *Ccn3*), which is a member of the CCN secreted ECM associated signaling protein family, also trended down in expression in 1% O_2_ in both Ctrl and cKO TFs, and cKO TFs in 1% O_2_ had reduced expression of *Scx*, although this was not significant (Fig. 7A). To define the extent to which hypoxia and *Hif1a* loss reshape TF transcriptional programs, we performed bulk RNA sequencing (RNA-seq) after 4 days in culture. In 1% O_2_, Ctrl TFs were enriched for genes associated with metabolic switching (Fig. 7B,C), including the HIF-1 signaling pathway (24 out of 708 differentially expressed genes; log_2_ fold change of 2.77), glycolysis and gluconeogenesis (19 genes; log_2_ fold change of 3.83) and the pentose phosphate pathway (11 genes; log_2_ fold change of 4.37). We also identified enrichment of molecular pathways associated with ECM deposition and remodeling, including osteoblast differentiation (*Osr2*, *Runx2*, *Smad6*, *Smad7*, *Smad9*, *Bmp4*, *Tgfbr1*, *Tgfbr2*), proteoglycans, and regulation of the actin cytoskeleton (Fig. 7B,C, Fig. S4).

In cKO TFs, *Casp12* was elevated compared to Ctrl regardless of oxygen availability, suggesting endoplasmic reticulum stress-induced apoptosis as a potential mechanism of cell death in the absence of *Hif1a* (Fig. 7D). In 1% O_2_, there were no differentially expressed caspase genes compared to 20% O_2_. Additionally, cKO TFs had upregulated expression of genes associated with the cell cycle, proliferation and survival including the long non-coding RNA *H19* and the apoptosis inhibitor *Bcl2* (Fig. 7D). Even in cKO cells, TFs cultured in 1% O_2_ had elevated genes associated with metabolism (e.g. *Slc40a1* and *Eno1*) compared to 20% O_2_, suggesting that *Hif1a* is not required for glycolytic and iron metabolism in hypoxia *in vitro* (Fig. 7D).

We also found that, in cKO TFs, the expression of Loxl and Col4 gene expression were reduced, as was *Myh9* and *Myh10*, suggesting a potential role for *Hif1a* in ECM crosslinking and cytoskeletal organization (Fig. 7D). *Bmp4* was upregulated in cKO TFs compared to Ctrl TFs, while the expression of Notch and Smad genes, *Runx2* and *Id1* were reduced (Fig. 7D). This suggests that cKO cells are prevented from fully differentiating into bone-forming osteoblasts (Cao et al., 2017; Dahlqvist et al., 2003). Despite this, cKO cells still generate a matrix consistent with disorganized pathogenic mineralization, indicating a process of calcification or heterotopic ossification (Cao et al., 2017; Dahlqvist et al., 2003).

### Hypoxic conditions *in vitro* lead to dysregulation in fibroblast metabolism and nascent matrix deposition regardless of genotype

**Fig. 8.**
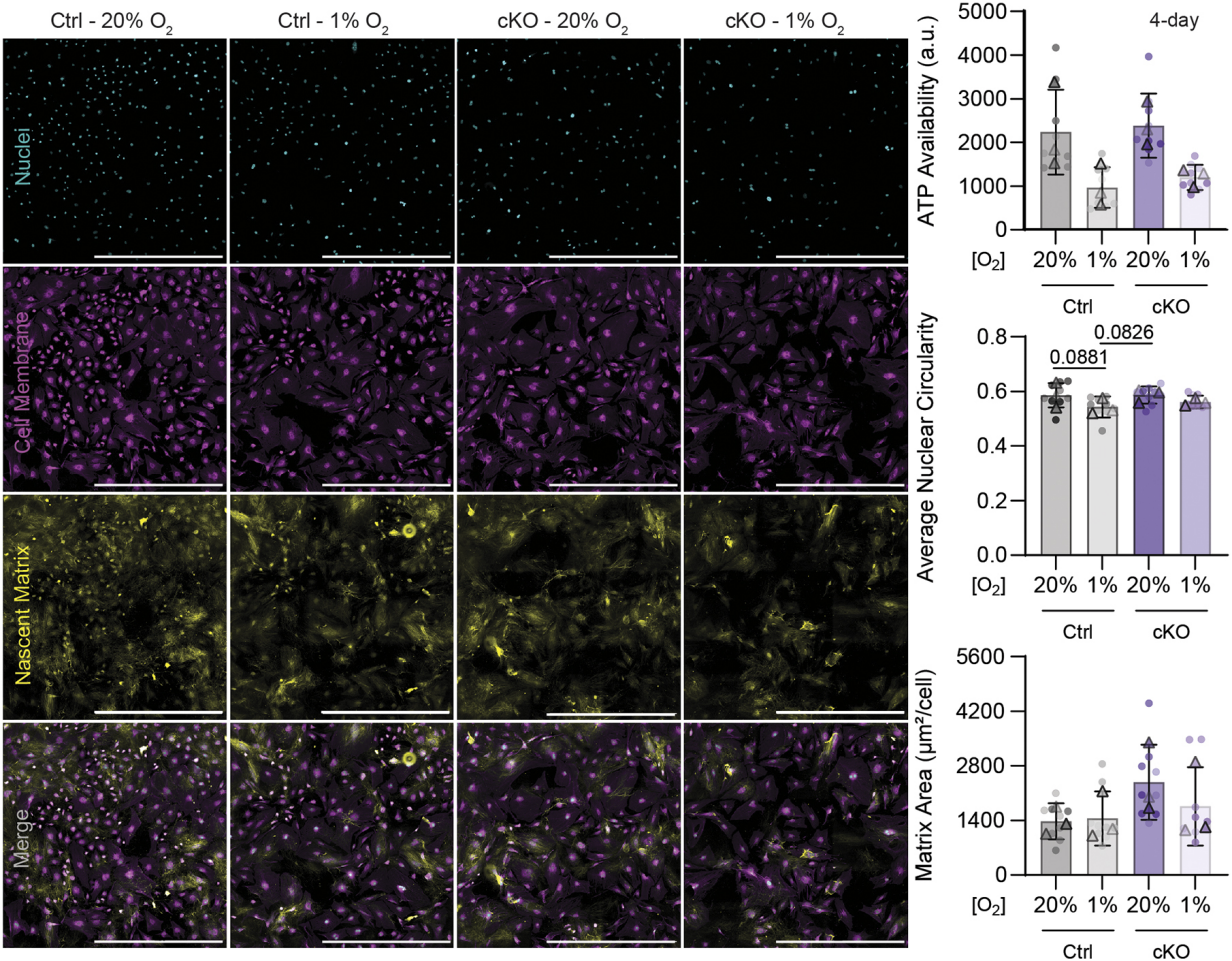
**Knockdown of *Hif1a in vitro* causes dysregulation in metabolism and nascent matrix deposition.** Nascent matrix staining of Ctrl and cKO TFs cultured in 20% O_2_ or 1% O_2_ for 4 days. Decreased ATP was available in hypoxic conditions regardless of genotype when normalized to cell density. Nuclear circularity trended down in Ctrl and cKO cells cultured in hypoxia compared to normoxia. Nascent matrix area may increase in cKO cells cultured in normoxia compared to other conditions when normalized to cell density. Bars show mean±s.d.; two-way ANOVA tests with Dunnett's multiple comparisons for hypoxia and genotype. Triangles denote average of one biological replicate across three technical replicates (*n*=3 per condition). a.u., arbitrary units. Scale bars: 1 mm.

After 4 days in culture, we observed decreased ATP availability in hypoxic conditions (1% O_2_) regardless of *Hif1a* expression (when normalized to cell density), supporting that TFs in hypoxia do not produce as much ATP as TFs in normoxia (20% O_2_) and instead rely on glycolysis (Fig. 8). Cell density did not significantly change at either the 2-day or 4-day time points, while ATP and matrix data at 2 days followed similar trends to 4-day cultures (Fig. S5A,C). Newly synthesized and secreted (nascent) matrix area was increased, although not significantly, in cKO cells cultured in normoxia compared to Ctrl cells, suggesting that cKO cells may generate more ECM under normoxic compared to hypoxic conditions (Fig. 8). Lastly, hypoxic conditions increased calcium, while glycosaminoglycan (GAG) production trended down in hypoxia at 2 weeks regardless of genotype (Fig. S5B). To determine what transcriptional differences may lead to the structural and mechanical defects seen in the cKO tendon-to-bone interface, we performed spatial transcriptomics on P5 Achilles entheses.

### Spatial transcriptomics at P5 reveals altered tendon–bone patterning in the cKO Achilles enthesis

**Fig. 9.**
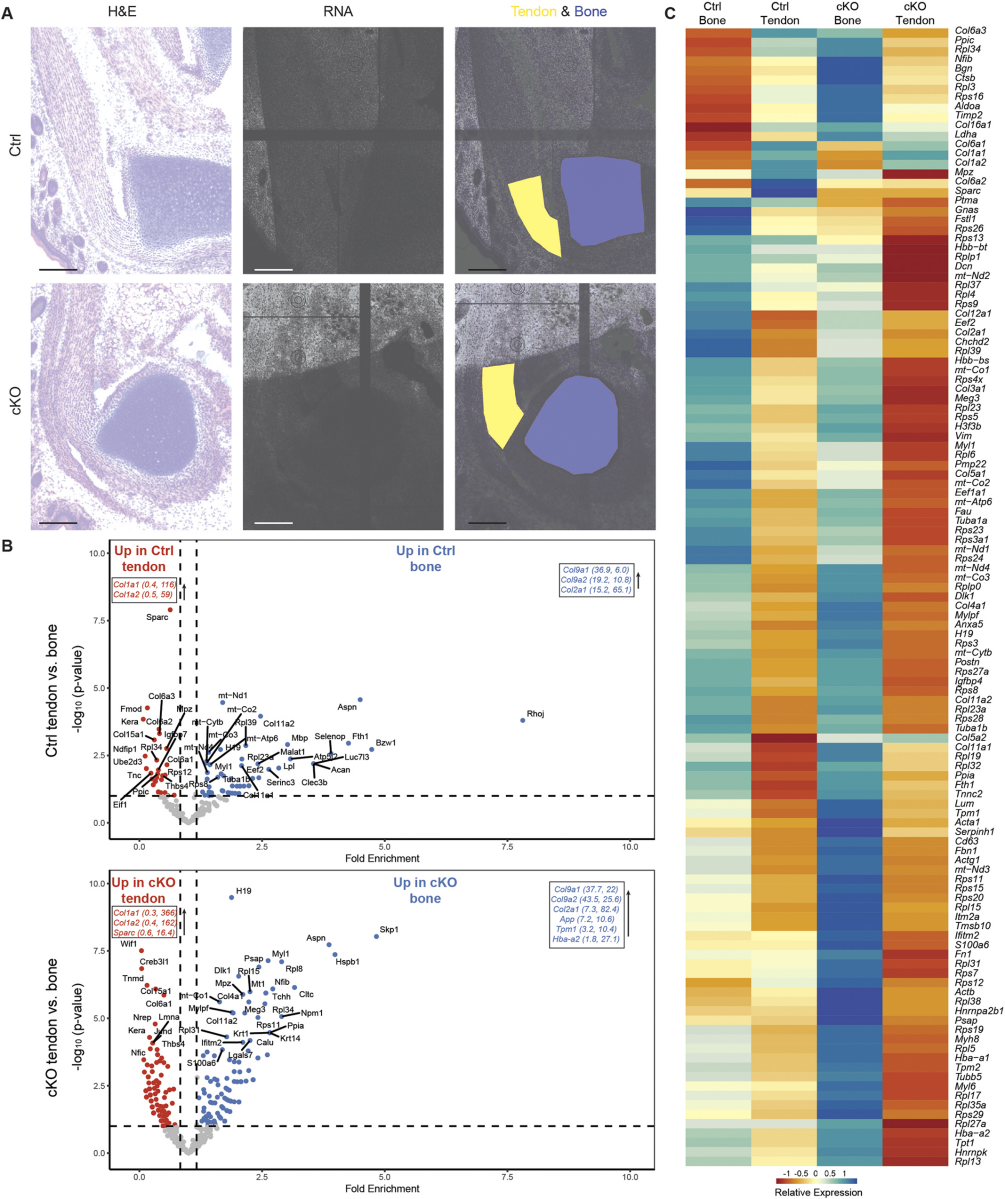
**Spatial transcriptomics at P5 reveals altered tendon–bone patterning in cKO animals.** (A) Hematoxylin and Eosin-stained sections of P5 mouse Achilles tendon insertions with transcripts overlapped and the tendon (yellow) and bone (blue) segmented. Scale bars: 200 μm. (B) Volcano plots indicating differentially expressed genes (DEGs) in Ctrl tendon versus bone comparison and cKO tendon versus bone comparison as defined by a gene with at least ten counts in either ROI, a *P*-value cutoff of 0.1, and a fold enrichment of at least 20%. Top 50 DEGs are labeled. (C) Heatmap of top DEGs, clustered using Ward's linkage normalized to the number of reads in each ROI. *n*=1 per genotype.

P0 cKO tendon-to-bone interfaces appeared to pattern similarly to Ctrl based on histology with defects emerging by P7 (Fig. 5A, Fig. S3). Therefore, sections from P5 Achilles and entheses were used to compare transcriptional differences between Ctrl and cKO mice that may lead to the dysregulation of cell and ECM organization. The spatial transcripts were first segmented into insertional tendon and bone regions (Fig. 9A). Segmented regions of tendon had higher reads with more unique genes compared to bone regions for both Ctrl and cKO sections, with these regions of interest (ROIs) yielded the following sequencing depths and detected genes: Ctrl bone (15,054 reads; 5032 unique genes), Ctrl tendon (21,368 reads; unique 5830 genes), cKO bone (13,287 reads; unique 4778 genes) and cKO tendon (38,494 reads; unique 7475 genes). Differential expression analyses were performed between tendon and bone ROIs within each section using a minimum inclusion threshold of greater than or equal to ten reads per gene. In general, when comparing relative expression between ROIs within samples, there were several ECM-associated genes that followed expected trends in expression between tendon and bone regardless of genotype, including *Dcn*, *Col1a1*, *Col1a2*, *Col6a1* and *Sparc* (elevated in tendon compared to bone in both Ctrl and cKO), *Col2a1*, *Col11a2*, *Col3a1*, *Postn* and *Aspn* (elevated in bone compared to tendon in both Ctrl and cKO) (Fig. 9C). However, a subset of genes represented divergent patterns of expression between tendon and bone that was dependent on genotype: for example, *Col6a3*, *Ppic*, *Timp2* and *Col5a1* had higher relative expression in Ctrl tendon compared to bone, yet cKO bone had elevated expression of these genes compared to cKO tendon (Fig. 9C). Conversely, *Mpz* and *Ctsb* were elevated in Ctrl tendon compared to Ctrl bone and was relatively lower in cKO tendon compared to cKO bone (Fig. 9C). Additionally, while some genes revealed no differences in expression between Ctrl bone and tendon (e.g. *Acta1*, *S100a6*, *Fn1*, *Psap*, *Serpinh1* and *Actb*), these genes were relatively elevated in cKO bone versus tendon. Only a few genes were relatively different between tendon and bone in cKO tissues, without these same genes showing obvious differences between Ctrl tendon and bone (*Col6a2*, *Ptma* and *Gnas*) (Fig. 9C). Together, these data support that Ctrl and cKO tendon/bone have overlapping and diverging transcriptional patterns, with many ECM genes that are classically associated with bone and tendon remaining unperturbed by *Hif1a* knockdown and other genes, primarily those associated with cell motility, signal transduction and cytoskeletal remodeling, being divergent in expression between tendon and bone in a *Hif1a-*dependent manner.

## DISCUSSION

This study reveals that the tendon enthesis maintains a hypoxic niche during early postnatal growth. This unique microenvironment that persists beyond birth and underlies key aspects of fibrocartilage development, cell survival and ECM organization. Using temporal mapping with EF5 staining, we showed that hypoxia is restricted to the mouse enthesis postnatally, while adjacent tendon and cartilage shifts towards higher oxygen concentrations soon after birth. This focal hypoxia highlights oxygen availability as a potential determinant of cell fate and ECM formation at the tendon–bone interface. Our findings build upon established parallels between the enthesis and the skeletal growth plate, both of which rely on hypoxia and HIF-1α for proper development (Blitz et al., 2013; Schipani et al., 2001; Amarilio et al., 2007; Bentovim et al., 2013; Schipani, 2010). However, unlike the growth plate which fuses upon skeletal maturity, the enthesis maintains an ‘arrested’ developmental state (Benjamin et al., 2002; Zelzer et al., 2014). In this study, we showed that loss of *Hif1a* in tendon and enthesis progenitors compromised enthesis cell survival during the postnatal growth, leading to impaired structural and cellular maintenance at the enthesis. This resulted in abnormal tendon–bone structural attachments, disrupted bone shape, and ultimately failure to maintain the fibrocartilaginous transition zone. These data suggest that maintaining HIF-1α is essential for the maintenance of a functional enthesis and is dispensable for tendon development and function.

The structural deficiencies we found in the enthesis and tendon are associated with underlying changes in TF biology. TFs are the primary architects of the collagen-rich tendon ECM, which is crucial for mechanical strength (Kannus, 2000). During fetal development, tendon-resident cells are highly proliferative, then shift to robust ECM production during postnatal growth (Felsenthal et al., 2018). In other mesenchymal populations, hypoxia induces a switch from oxidative phosphorylation and mitochondrial respiration to glycolysis, a pathway essential for cell survival under low oxygen conditions (Schito and Rey, 2018). Chondrocyte survival in growth plate hypoxia, for instance, relies on the suppression of mitochondrial respiration (Dyment et al., 2015; Breidenbach et al., 2015; Yao et al., 2019). We characterized the metabolic shifts typically associated with hypoxia in cells derived from tendon. Our transcriptomic profiling of TFs revealed that *Hif1a*-deficient cells were less capable of executing these metabolic switches, supporting the idea that hypoxic adaptation in the tendon requires HIF-1α. Interestingly, despite classical concepts, we did not observe increased TF proliferation or enhanced ECM deposition *in vitro* under hypoxic conditions, suggesting that TF responses to hypoxia may be more nuanced, and perhaps dependent on additional factors. Further, our spatial transcriptomic data unveiled a possible dysregulation of cell proliferation, apoptosis, and growth pathways in which the cells may be signaled to proliferate but are failing during the cell cycle due to DNA damage, possibly leading to the dysregulation of cell population homeostasis. These spatial data should be interpreted as exploratory. Because the analysis was ROI-based with limited replication and read depth varying across ROIs, and because comparisons were performed primarily within sections (tendon ROI versus bone ROI within a tissue sample), the dataset is not well powered for definitive genotype-specific, tendon-intrinsic conclusions. Instead, these results nominate candidate pathways for validation in follow-up studies (e.g. bulk RNA-seq of isolated compartments and/or higher-replicate spatial approaches). In tandem with increased TUNEL staining in the developing cKO enthesis, these transcriptional findings indicate that knockdown of *Hif1a* in tendon-resident cells disrupts cell-cycle and apoptosis pathways, likely contributing to the structural and mechanical defects observed during tendon and enthesis development. Notably, these signals are likely spatially and temporally heterogeneous as apoptosis appears to be enriched in the developing enthesis, whereas increased cell density was observed later in the insertional tendon. This may reflect compensatory or maladaptive remodeling, consistent with an overall dysregulation or imbalance of proliferation and apoptosis.

Pathologically, the disrupted cellular environment at the cKO enthesis may promote recruitment of macrophages and other immune cells, compounding tissue damage and degeneration. Elevated HIF-1α in tendinopathy is associated with proinflammatory cytokine production and apoptosis, contributing to ECM breakdown and poor healing outcomes (Millar et al., 2012). Other oxygen tension-dependent mechanisms, such as the modulation of *Rac1* activity, have also been implicated in tendon disorders, reinforcing the clinical relevance of these developmental mechanisms (McBeath et al., 2019). Further, the developing tendon and enthesis may experience a different oxygen environment than mature tendon (Howell et al., 2017), and it is important to note that, while HIF-1α can be induced by hypoxia, HIF-1α expression can also be induced by inhibiting the prolyl-4-hydroxylase domain (PHD) enzymes, which leads to the stabilization of HIF-1α (Jaśkiewicz et al., 2022). For example, transforming growth factor β1 (TGFβ1) can inhibit PHD expression and stabilize HIF-1α in fibrosarcoma cell lines and mouse embryonic fibroblasts (McMahon et al., 2006; Lee et al., 2019). As such, we found oxygen-independent mechanisms by which HIF-1α may also be regulated in the tendon and enthesis.

The observed modifications in gene expression patterns of TFs *in vitro* further support the hypothesis that HIF-1α modulates metabolic switches, ECM synthesis and cytoskeletal organization under low oxygen tension. Specifically, the blunted transcriptional and metabolic responses of *Hif1a*-deficient TFs to hypoxia, along with changes in ECM-related gene expression, suggest that adaptation to hypoxia is not solely compensatory but may be required for proper ECM assembly and maintenance. We showed that the ability of TFs to deposit ECM may be dependent on *Hif1a*, and we have identified potential regulators of cell function including cell cycle and ECM regulation that were independent of hypoxia. The upregulation of apoptotic markers and alterations in cell cycle-related genes hint at possible mechanisms for impaired tissue integrity in the absence of *Hif1a*. As we used cells isolated from tail tendons for tissue culture experiments, which are positional and not load bearing like the Achilles tendon, these cells may not fully recapitulate the microenvironment or cellular responses of the load-bearing Achilles enthesis. Additional studies using cells derived from the mouse Achilles tendons or other load-bearing tendons could better validate these findings.

While our study focuses on tendon-resident Hif1a function using constitutive *Scx*Cre, it is important to recognize that *Hif1a* loss in broader limb mesenchymal populations can produce primary defects in endochondral skeletal development. In particular, *Prrx*1Cre–mediated ablation of *Hif1a* (in the limb bud mesenchyme) causes marked growth plate abnormalities and impaired chondrogenesis, underscoring the requirement for HIF-1α during cartilage and growth plate maturation in the embryonic limb (Schipani et al., 2001). This prior work is relevant to interpretation of *Scx*Cre models because constitutive *Scx*Cre does not perfectly restrict recombination to TFs; *Scx* expression is developmentally dynamic and is expressed in the skeletal primordia (Asou et al., 2002) and condensing cartilage (Cserjesi et al., 1995), and recombination in non-tendon cells (Wernlé et al., 2023) likely contributes to the phenotype we observed. Consequently, some components of the tendon and enthesis phenotype could reflect a combination of tendon- and enthesis-intrinsic effects of *Hif1a* loss and secondary consequences of altered growth plate maturation. This is particularly relevant because disruptions in growth plate development can alter long-bone growth, joint and epiphyseal geometry, and local mechanical loading, which in turn may influence enthesis loading and mineralization trajectories during postnatal maturation. In the future, more targeted gene knockdown using inducible, tendon-specific *Cre* lines will be worth pursuing.

Taken together, our results indicate hypoxia as a defining feature of the developing enthesis, with HIF-1α functioning as a gatekeeper of cell survival, ECM organization and tissue architecture at the tendon–bone interface. These discoveries have important implications for understanding enthesopathies, such as those seen in rotator cuff injuries, where impaired ECM organization and cell death are observed (Fu et al., 2021). Furthermore, these data suggest that maintaining a hypoxic niche or manipulating HIF-1α signaling could serve as a therapeutic or tissue engineering strategy to promote functional enthesis regeneration.

The maintenance of a hypoxic niche at the postnatal enthesis and the necessity of HIF-1α for cell survival and structural organization establish new principles underlying the development of the tendon-to-bone interface. Our results open avenues for further research and ultimately inform potential therapies for tendon and enthesis repair through the manipulation of oxygen in the interfacial environment to further influence the guided regeneration of the cellular and ECM gradients necessary for mechanical function post-injury.

## MATERIALS AND METHODS

### Mouse husbandry

*Hif1a^flox/flox^; ScxCre* mice (C57BL6J/129S1 mixed background) were generated by crossing male *Hif1a* heterozygous (*Hif1a^flox/WT^*) *ScxCre* mice with homozygous *Hif1a^flox/flox^* females to generate constitutive knockout mice (cKO; *Hif1a^flox/flox^*;*ScxCre*) and Ctrl mice (Cre-negative *Hif1a^flox/flox^* or *Hif1a^flox/WT^*;*ScxCre*). All experiments were approved by institutional review (IACUC). Mice were euthanized following IACUC approvals (carbon dioxide asphyxiation if >8 days of age or decapitation if <8 days of age). When possible, left and right limbs from each mouse were used in complementary assays (e.g. histology/nanoCT using left limbs and biomechanical testing using right limbs) to reduce animal numbers. A total of 156 mice, including Ctrl, were used across all time points.

### EF5 staining

Because of the intrinsic link between hypoxia and *Hif1a*, we first determined whether a hypoxic environment exists in the developing Achilles enthesis using EF5 staining, which in the presence of hypoxia forms cellular adducts that can be visualized by immunostaining (*n*≥3 C57BL6J embryos or mice per time point) (Koch, 2002). 2-Nitroimidazole EF5 compound (10 mM) was administered intraperitonially in timed-pregnant C57/BL6J dams (E16.5) or subcutaneously in neonatal pups 2 h prior to euthanasia (at P1 and P5). Hindlimbs were embedded in optimal cutting temperature (OCT) glycol-based embedding media, cryosectioned, and stained with ELK3-51 AF488 antibody (75 μg/ml) with and without competed stains (EF5 Hypoxia Detection Kit, Alexa Fluor 488; Sigma-Aldrich, EF5-30A4M). Cells experiencing hypoxic stress accumulated EF5 adducts and were visualized using fluorescence microscopy (ECLIPSE Ni-U, Nikon; 20×). Tissues were counterstained with DAPI for nuclear staining.

### Gross anatomy, X-ray imaging, and forelimb grip strength

Ctrl and cKO mice were weighed at 4 or 8 weeks of age (*n*=15 for Ctrl and *n*=11 for cKO at 4 weeks of age; *n*=13 for Ctrl and *n*=8 for cKO at 8 weeks of age) and grip force was measured using a grip strength test device (BIO-GS4, Bioseb). Mice were then euthanized to visualize gross musculoskeletal anatomy and skeletal morphology using planar X-ray (Faxitron MX-20; 25 kV, 8 s; digitized with Fujifilm FCR Prima T2).

### Tendon and enthesis biomechanics

After euthanasia, intact left limbs from Ctrl (Cre-negative, *n*=15; Cre-positive, *n*=3 males) and cKO mice (*n*=15) at 8 weeks of age were frozen at −20°C. Achilles tendons and calcaneus were dissected and kept hydrated (with removal of the plantaris tendon and surrounding muscles). Tendon volume was imaged using OCT with polarization (TEL221PSC1, OCT-LK4 lens, ThorLabs Inc.). Intensity and polarization images were acquired in 3D (A-scan averaging=4; spacing=10 μm in *x* and *y*, 2.5 μm in *z*; sampling rate=28 kHz) and intensity and retardation images were exported (32-bit TIFF). The full 3D OCT image stacks were analyzed on a slice-level using a 2D FFT method to measure frequency of retardation, which describes collagen organization (MATLAB). For each slice, we extracted the distance from the center to the maximum peak in frequency space by summing the pixel values in 2-pixel-wide concentric rings radiating from the center. The AUC of dominant frequency versus slice-depth curve is reported per sample as collagen organization metric where a higher retardation frequency (i.e. more tightly packed retardation patterns) results in a higher AUC after FFT, implying increased organization. Slice analysis was used to determine tendon CSA and bifurcation distance (Dragonfly, Comet Technologies Canada Inc.). After imaging, the proximal Achilles tendon was clamped in a thin film grip (Imada) with the calcaneus secured in a custom 3D-printed fixture (FormLabs 3B, Somerville, MA, USA) prior to uniaxial tensile testing. The tendon and grip were placed in a room temperature PBS bath and a pin was used to secure the tendon-grip unit. Uniaxial tensile tests were conducted with load measured using a multi-axis load cell (±70 N; Mach-1 VS500CST, Biomomentum). Samples were preloaded to 0.2 N and preconditioned for ten cycles (±0.025 mm at 0.1 Hz). Grip-to-grip gauge length (L_0_) was measured at the start of each test after preload and was consistent between tests (3.6±0.5 mm). Load and torque data were collected while applying displacement to failure at 0.01 mm/min to assess axial and off-axis loading. Mechanical properties (e.g. maximum load, maximum strain, linear stiffness; linear modulus and maximum stress calculated using load and CSA) of the Achilles tendon were calculated from force-displacement data using a piecewise linear segmentation by dynamic reprogramming recursion package (dpseg) in R (v4.2.2 or later; The R Project for Statistical Computing, Vienna, Austria). Data are presented as mean±s.d. and compared using a one-way ANOVA and corrected for multiple comparisons using Tukey's multiple comparisons tests (Prism v9.0+; GraphPad).

### NanoCT

Right mouse hind limbs at 8 weeks of age were fixed at 4% paraformaldehyde. After fixation, limbs were suspended in a 3% agar solution and scanned using a Nantom M scanner (nanoCT; 8 μm voxel resolution; 80 kV, 400 μA, 0.381-mm aluminum filter, 500-ms timing with three averaged frames and one skipped frame after axis rotation; 2000 projection images acquired at a source-to-axis distance of 48 mm; Waygate Technologies LP). 3D reconstructed nanoCT images were analyzed using Dragonfly software (v2021.3; Object Research Systems, Inc.) to measure calcaneal bone morphometry. Data are presented as mean±s.d. and compared using a one-way ANOVA and corrected for multiple comparisons using Tukey's multiple comparisons tests (Prism v9.0+; GraphPad). For shape analysis of the calcanei, nanoCT images were filtered in Dragonfly using Laplacian smoothing (60 iterations) and STL files were imported into ShapeWorks Studio (Cates et al., 2017). Shapes were groomed to fill gaps and aligned based on anatomical landmarks and optimization (128 particles; initial relative weighting=0.25; relative weighting=75; starting/ending regularization=1000; 1000 iterations per split; optimization iterations=2000; Procrustes rotation/translation; Procrustes interval=10; narrow band=4).

### Histology, TUNEL staining and TEM

Hind limbs from Ctrl and cKO mice at P0, P7, P14 and P56 (8 weeks of age) were decalcified in 14% ethylene diamine tetra-acetic acid (EDTA, pH 7.2) after fixation and/or nanoCT then paraffin embedded for histology. Tissues were embedded in the sagittal plane to visualize the Achilles enthesis and tendon midsubstance and sectioned at 5-10 μm and serial slides were stained for Hematoxylin and Eosin, Toluidine Blue, Safranin O, silver nitrate or Picrosirius Red. Slides were imaged in brightfield using a 20× objective on a Cytation 10 (Agilent). Sections stained with Picrosirius Red were imaged using circular polarized light microscopy with a 10× objective on an epifluorescent microscope (Leica) and a polarization camera and ThorCam software (ThorLabs Inc.) for quantitative polarized light microscopy. The degree of linear polarization and the standard deviation of angle of polarization were analyzed using the Math and SciPy Stats libraries in Python3. Data were compared using two-way ANOVA between genotype and age (Prism GraphPad, v10). Paraffin sections from Ctrl and cKO mice at P7 and P14 were stained using the In Situ Cell Death Detection Kit (Roche, 11 684 817 910) with DAPI (to stain nuclei). TUNEL-stained slides were imaged using epifluorescence microscopy (ECLIPSE Ni-U, Nikon, or Cytation 10). For TEM, Achilles tendons from adult (8 weeks old) Ctrl and Hif1a cKO mice were fixed with glutaraldehyde and osmium tetroxide and were embedded in plastic (*n*=2/group). Ultrathin sections were produced to visualize the ultrastructure and collagen fibrils using a JEOL-1400 transmission electron microscope (5000×, NANOSPRT12 camera, 960 ms exposure×2std. frames, Gain:1, Bin: 1). Images were analyzed using Fiji/ImageJ using Otsu thresholding, watershed segmentation, and particle analysis (Schneider et al., 2012).

### *In vitro* TF culture for gene expression and cell assays

Ctrl (Cre-negative) and cKO mouse tail TFs were isolated from 4- to 6-month-old male and female mice (*n*=3) for *in vitro* studies using collagen gels (PureCol-S, Millipore Sigma) (Kent et al., 2022) TFs from Ctrl and cKO mice were seeded on collagen-I-coated-tissue-culture plastic at 2500 cells/cm^2^ in DMEM:F12 containing 1% fetal bovine serum and 1% Penicillin/Streptomycin in either 20% O_2_ or 1% O_2_ conditions (Whitley H35 Hypoxystation, Don Whitley Scientific). Total RNA was isolated after 1 week in culture [for quantitative polymerase chain reaction (qPCR)] or 4 days in culture (for bulk RNA-seq) using spin-columns (PureLink RNA mini kit, Thermo Fisher Scientific) with on column genomic DNA digestion (RNase-free DNase, QIAGEN). RNA quality and quantity were checked using a NanoDrop spectrophotometer (qPCR; Thermo Fisher Scientific) or Bioanalyzer (RNA-seq; Agilent 2100 Bioanalyzer). RNA with a 260/280 ratio greater than 2.0 was reverse transcribed to cDNA (qPCR; SuperScript IV VILO Master Mix, Thermo Fisher Scientific) and 10 ng/μl of cDNA was used per reaction. Quantitative real-time reverse transcription polymerase chain reaction (qRT-PCR) was performed in triplicate for each gene (Table S1) using a CFX96 Real-Time PCR Detection System (Bio-Rad) with Power SYBR Green PCR Master Mix (Thermo Fisher Scientific) with *Rplp0* and *Polr2a* were used as reference genes. After normalization to reference gene Cq values, fold change data were compared using two-way ANOVAs and corrected for multiple comparisons with Tukey's multiple comparisons tests (Prism v9.0+; GraphPad).

For bulk RNA-seq (4 days in culture), samples (RNA Integrity Number>8.6) were submitted for Poly-A enrichment library preparation (Illumina) and sequenced using 151 bp paired-end sequencing (Illumina NovaSeqXPlus, System Suite v1.3.0.39308). De-multiplexed Fastq files were generated using BCL Convert Conversion Software v4.0 (Illumina). Cutadapt v4.8 (Martin, 2011) was used to trim reads and FastQC v0.11.8 was used to ensure data quality (https://www.bioinformatics.babraham.ac.uk/projects/fastqc/; accessed 2 July 2025). Next, Fastq Screen v0.15.3 was used to screen for contamination (Wingett and Andrews, 2018). Reads were then mapped to the reference genome GRCm38 (ENSEMBL) using STAR v2.7.8a (Dobin et al., 2013) and assigned count estimates to genes with RSEM v1.3.3. (Li and Dewey, 2011). Alignment options followed ENCODE standards for RNA-seq. Multiqc v1.20 compiled the results from several of these tools and provided a detailed and comprehensive quality control report (Ewels et al., 2016). Differential gene expression was determined from count matrices with a paired design (e.g. Cre-negative Ctrl versus cKO) in DESeq2 in R/Bioconductor (Love et al., 2014). We used Database for Annotation, Visualization, and Integrated Discovery (DAVID) to analyze biological processes and Kyoto Encyclopedia of Genes and Genomes (KEGG) pathways (Sherman et al., 2022; Huang et al., 2009).

Additionally, mouse TFs from Ctrl and cKO mice were cultured for 2 or 4 days *in vitro* and ATP availability was measured in white opaque 96-well TC-treated plates (Falcon, 353296) using CellTiter GLO 2.0 Cell Viability Assay (luminescence; Promega) per manufacturer recommendations. Data were analyzed using a two-way ANOVA with Šidák's multiple comparisons for genotype and oxygen concentration (*n*=3 biological replicates with one male and two female for Ctrl and two male and one female for cKO; *n*=3 technical replicates per biological replicate). On parallel plates (96-well, black-walled, glass-bottom; CellVis, P96-1.5P), TFs were administered nascent metabolic protein labeling media (Loebel et al., 2022) with 1% fetal bovine serum (replenished every 48 h) to label nascent matrix deposited by TFs. After 4 days in culture, TFs were placed at 4°C for 5 min, washed with cold 1× Dulbecco's phosphate buffered solution (DPBS) then with cold 2% bovine serum albumin solution, and treated with 15 μM DBCO-488 for 20 min at 4°C. Following this, TFs were washed with cold 2% bovine serum albumin and fixed in 4% paraformaldehyde. TFs were stained with 0.1% CellMask Orange and Hoechst 33342 (Invitrogen, H3570) to label the cell membrane and nuclei, respectively. Lastly, TFs were imaged using spinning disk confocal microscopy at 20× strength on a Cytation 10 (Agilent). Images were processed and analyzed using Gen5 (Agilent) to quantify nuclear size/shape and nascent matrix area. Data were compiled in GraphPad (Prism v9.0+; GraphPad) and analyzed with two-way ANOVAs with Šidák's multiple comparisons for genotype and oxygen concentration. Also, in parallel, Ctrl and cKO cells were cultured in 20% O_2_ normoxia or 1% O_2_ hypoxia for 2 weeks and total GAG and calcium content from cell lysates were analyzed using a total glycosaminoglycan kit (abcam, ab289842), and a calcium assay kit (Sigma-Aldrich, MAK477), respectively.

### Spatial sequencing library preparation and analysis

All spatial sequencing data were generated using a recently published Seq-scope protocol (Kim et al., 2025). In brief, glycol-embedded P5 mouse hindlimbs were flash-frozen in liquid nitrogen and stored at −80°C until sectioning. Sections (10 μm) were mounted onto a Seq-Scope Chip, processed by Illumina NovaSeq 6000 flow cell, and fixed in cold methanol. Tissues were stained with Hematoxylin and Eosin and imaged using a light microscope (Keyence). Tissues were permeabilized with pepsin for 24 min to release mRNA onto the chip. The library was generated and sequenced as described in the protocol (Kim et al., 2025). Data analysis was performed using a standard ‘novascope’ pipeline as described in the protocol paper (Kim et al., 2025) to produce a spatially indexed digital gene expression map (sDGE). Using sDGE and the polygonal coordinates drawn upon a histology image matching the sDGE, we segmented the insertional tendon and bone areas and extracted gene expression from the segments based on the spatial coordinates. Using the digital gene expression data, within-sample comparisons of tendon and bone (Ctrl and cKO samples) were performed as well as between-sample comparisons (Ctrl versus cKO tendon; Ctrl versus cKO bone). Differentially expressed genes were identified as genes with at least ten reads in a sample with a *P*-value of 0.1 in comparisons within a section. Across sections, a fold enrichment of 20% was used to identify differentially expressed genes. Counts were normalized to the number of reads within each ROI drawn. We used the DAVID to analyze biological processes and KEGG pathways and R to analyze these data.

## Supplementary Material



10.1242/develop.205458_sup1 Supplementary information
