# Supplementary material for: The developing tendon and enthesis are hypoxic and rely on hypoxia-inducible factor 1a during postnatal development
Source: Development. 2026 Jun 22;153(16):dev205458. doi: 10.1242/dev.205458 (PMC13354958; doi:10.1242/dev.205458)
Supplement: Supplementary information [file develop-153-205458-s1.pdf]

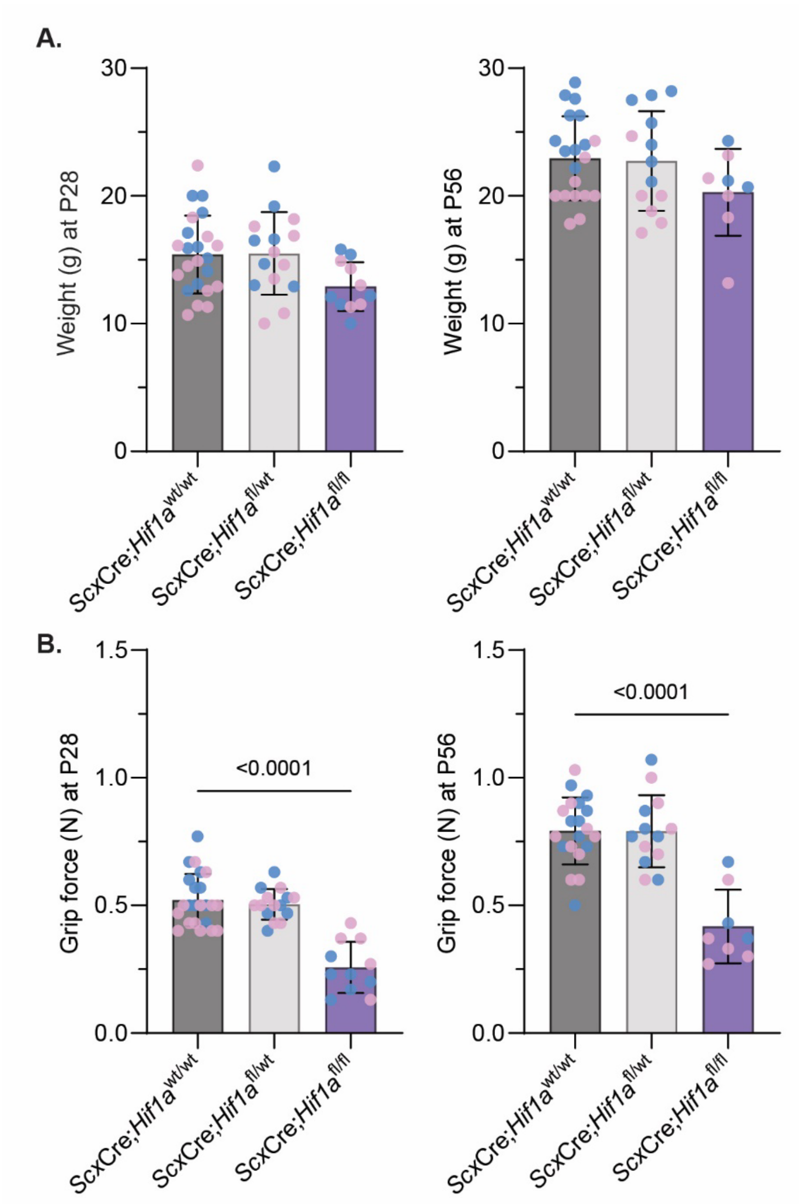

Fig. S1. Weight is comparable across genotypes while grip force is reduced in *Hif1a* knockout animals. (A) Homozygous wild-type and heterozygous animals have comparable weights at P28 and P56. *Scx*Cre; *Hif1a*$^{wt/wt,}$ N=10 male and N=13 female; *Scx*Cre; *Hif1a*$^{fl/wt,}$ N=7 male and N=8 female; *Scx*Cre; *Hif1a*$^{fl/fl,}$ N=6 male and N=5 female.

(B) Grip force is reduced in *Hif1a* knockout animals compared to homozygous and heterozygous controls and both P28 and P56 while homozygous wild-type and heterozygous animals have comparable grip forces at P28 and P56. *Scx*Cre; *Hif1a*$^{wt/wt,}$ N=10 male and N=13 female; *Scx*Cre; *Hif1a*$^{fl/wt,}$ N=7 male and N=8 female; *Scx*Cre; *Hif1a*$^{fl/fl,}$ N=6 male and N=5 female.

Development • Supplementary information

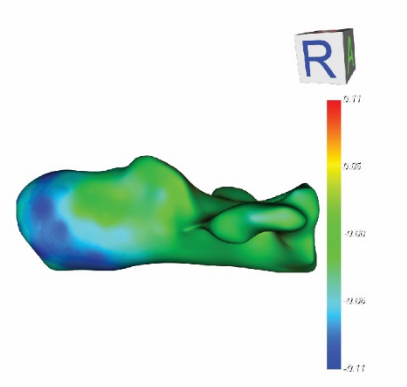

Mode	Eigen Value	Explained Variance	Cumulative Variance
1	0.308772	51.10%	51.10%
2	0.0714029	11.80%	62.90%
3	0.0420211	7.00%	69.90%
4	0.0391044	6.50%	76.40%
5	0.0287754	4.80%	81.10%
6	0.0267873	4.40%	85.50%
7	0.0251313	4.20%	89.70%
8	0.0185613	3.10%	92.80%
9	0.0146022	2.40%	95.20%
10	0.0107796	1.80%	97%
11	0.00747381	1.20%	98.20%
12	0.00445566	0.70%	99.00%
13	0.00340519	0.60%	99.50%
14	0.00291007	0.50%	100.00%

Fig. S2. Most of the differences in calcanei shape are due to changes near the tendon-to-bone interface. Modes 1 and 2 account for over 60% of the change in shape. Ctrl (Cre-negative), N=6 males and N=2 females; cKO, N=4 males and N=6 females.

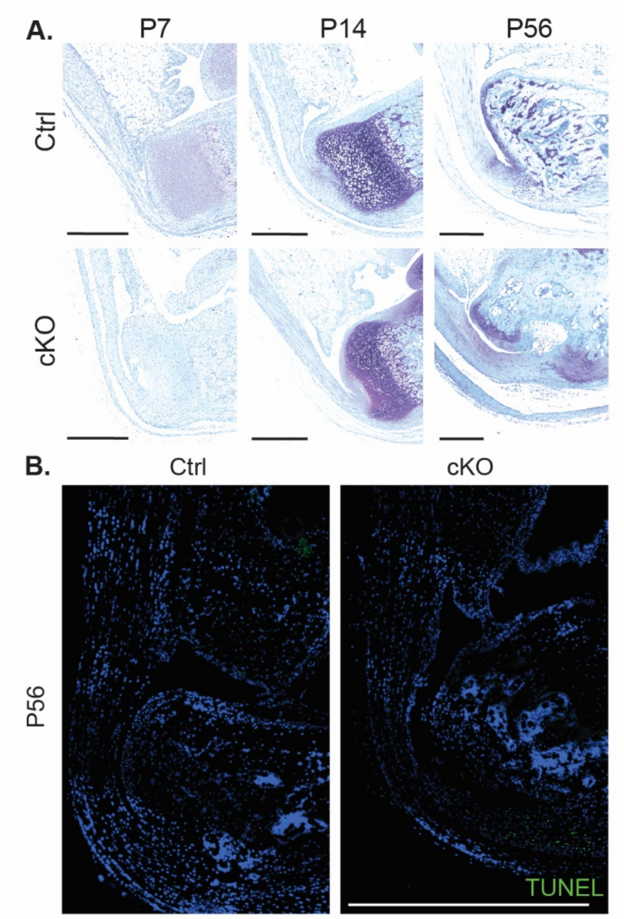

Fig. S3. Enthesis defects present during postnatal development. (A) Hematoxylin and Eosin staining at P0 show unremarkable differences due to *Hif1a* knockout while disorganized cell and ECM are present at P7 and P56 entheses. Ctrl (Cre-negative), N=3 per timepoint; cKO, N=3 per timepoint. (B) TUNEL staining at P56 revealed no apoptotic cells in the tendon and enthesis of Ctrl and cKO mice while some TUNEL positive cells were present at the base of the calcaneus in cKO mice. Ctrl (Cre-negative), N=3; cKO, N=3.Scalebar = 1mm.

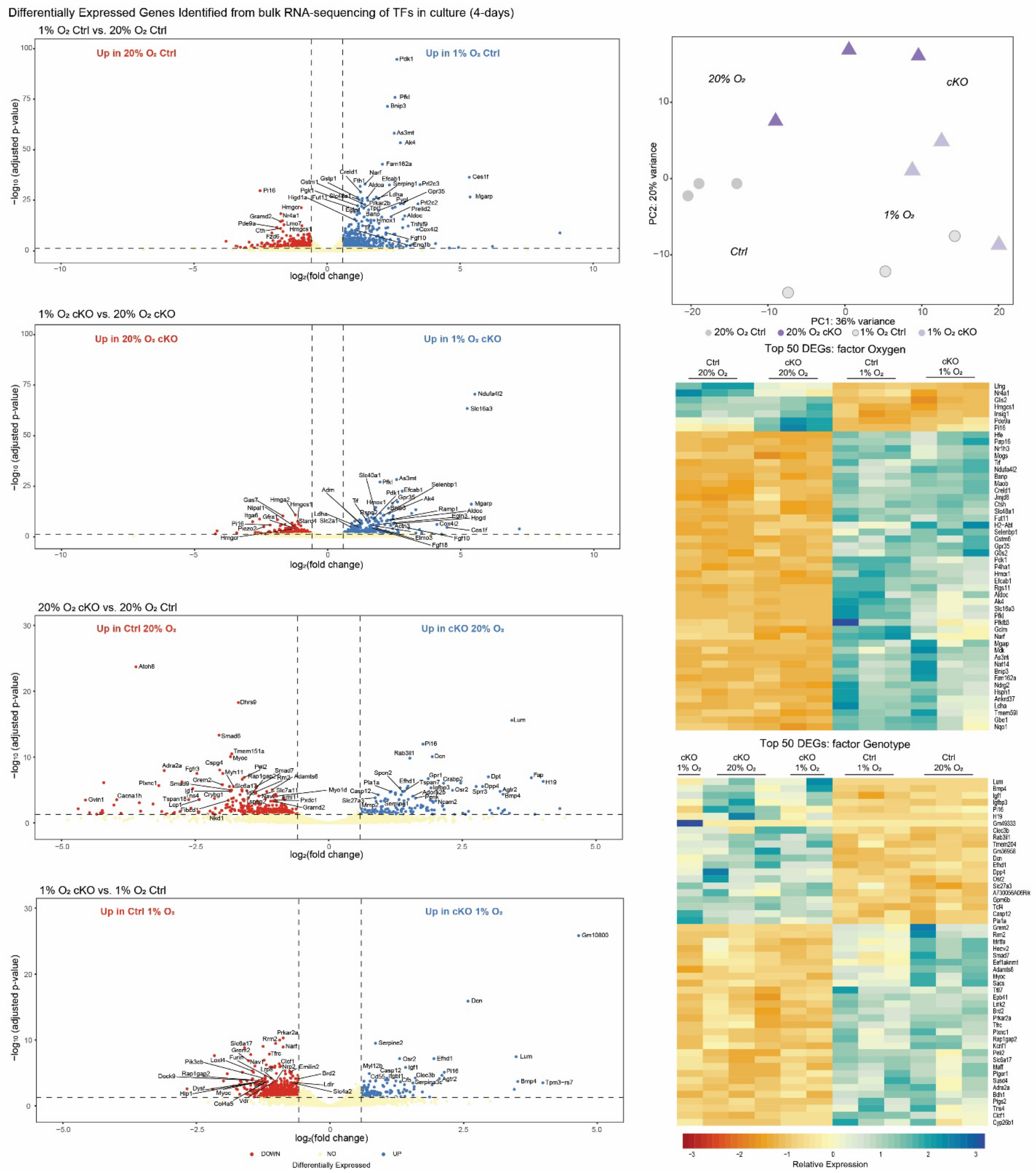

Fig. S4. Top 50 differentially expressed genes across all comparisons in 4-day RNA-sequencing data of *in vitro* cell cultures. N=3 per condition.

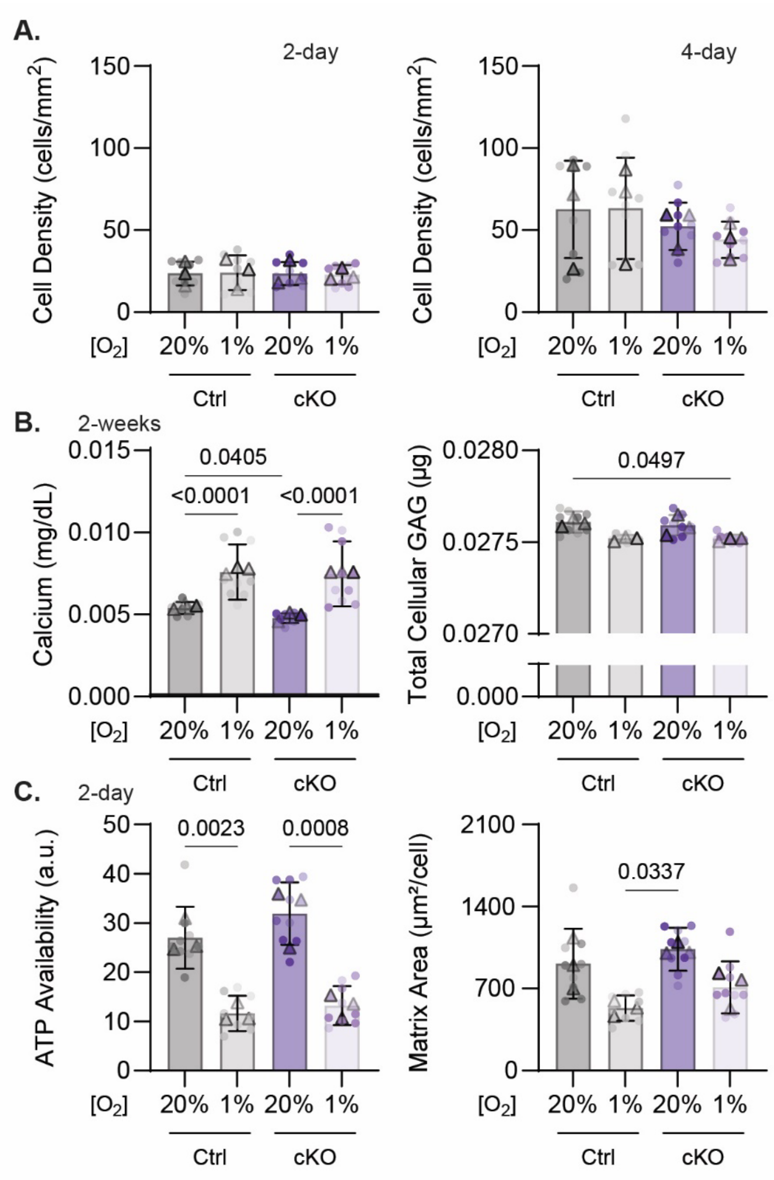

Fig. S5. Hypoxic conditions yield increased calcium and reduced GAG, ATP availability, and matrix area. (A) Cell density did not change at either 2- or 4-days in cell culture across conditions while initial seeding density was similar. (B) Calcium available in solution is increased in hypoxic 4-day cultures while GAG from cell and matrix lysates is reduced at 2-weeks. (C) 2-day cultures show similar trends in ATP availability and matrix area as 4-day data with reduced ATP available and nascent matrix deposited in hypoxic conditions regardless of genotype (Figure 8). Triangles denote average of one biological replicate across 3 technical replicates. N=3 per condition.

Table S1. Integrated DNA Technologies (IDT) PrimeTime qPCR Primer Assay IDs

Gene	Assay ID
Rplp0	Mm.PT.58.43894205
Polr2a	Mm.PT.39a.22214849
Hif1a	Mm.PT.58.11211292
Hilpda	Mm.PT.42333419
Nov (Igfbp9)	Mm.PT.58.33406873
