# Supplementary material for: The developing tendon and enthesis are hypoxic and rely on hypoxia-inducible factor 1a during postnatal development
Source: Development. 2026 Jun 22;153(16):dev205458. doi: 10.1242/dev.205458 (PMC13354958; doi:10.1242/dev.205458)
Supplement: Supplementary file 1 [file develop-153-205458_review_history.pdf]

Development

The developing tendon and enthesis are hypoxic and rely on hypoxia-inducible factor 1a during postnatal development

Stephanie S. Steltzer, Nicole Migotsky, Tessa Phillips, Syeda N. Lamia, Bachir A. Abeid, Kiwon Lee, Seung-Ho Bae, Connor Leek, Sydney Grossman, Moaid Shaik, Allison Risha, Kaitlyn Frey, Claudia Loebel, Jun Hee Lee, Yatrik Shah, Adam C. Abraham and Megan L. Killian

DOI: 10.1242/dev.205458

Editor: Alex Hughes

Review timeline

Original submission:	17 December 2025
Editorial decision:	25 January 2026
First revision received:	14 April 2026
Accepted:	19 May 2026

Original submission

First decision letter

MS ID#: dev.205458

MS TITLE: The developing tendon and enthesis are hypoxic and rely on hypoxia-inducible factor 1a (Hif1a) during postnatal development

AUTHORS: Stephanie S. Steltzer; Nicole Migotsky; Tessa Phillips; Syeda N. Lamia; Ki won Lee; Sueng-Ho Bae; Connor Leek; Sydney Grossman; Moaid Shaik; Allison Risha; Kaitlyn Frey; Claudia Loebel; Jun Hee Lee; Yatrik Shah; Adam C Abraham; Megan Leigh Killian

Dear Dr Killian,

I have now received all the referees' reports on the above manuscript, and have reached a decision. The referees' comments are appended below.

As you will see, the referees express considerable interest in your work, but have some significant criticisms and recommend a substantial revision of your manuscript before we can consider publication. If you are able to revise the manuscript along the lines suggested, which may involve further experiments, I will be happy to receive a revised version of the manuscript. Your revised paper will be re-reviewed by one or more of the original referees, and acceptance of your manuscript will depend on your addressing satisfactorily the reviewers' major concerns. Please also note that Development will normally permit only one round of major revision. If it would be helpful, you are welcome to contact us to discuss your revision in greater detail. Please send us a point-by-point response indicating your plans for addressing the referees' comments, and we will look over this and provide further guidance.

Please attend to all of the reviewers' comments and ensure that you upload both a 'clean' version of your Word file, along with a highlighted version clearly showing where you have made changes in the revised manuscript. Please avoid using 'Tracked changes' in Word files as these are lost in PDF conversion. I should be grateful if you would also provide a point-by-point response detailing how you have dealt with the points raised by the reviewers in the 'Response to Reviewers' box. If you do not agree with any of their criticisms or suggestions please explain clearly why this is so.

© 2026. Published by The Company of Biologists under the terms of the Creative Commons Attribution License (https://creativecommons.org/licenses/by/4.0/).

Reviewer 1

SUMMARY OF THE ADVANCE MADE IN THIS PAPER AND ITS POTENTIAL SIGNIFICANCE TO THE FIELD

This manuscript from Steltzer et al., is focused on the role of Hif1a during post-natal development of the tendon and enthesis. Overall, the manuscript is well-written and the data largely support the conclusions. The authors are commended on the very nice qPLI data and the relatively robust characterization of Hif1a conditional knockout animals. The primary limitation of the study is the overreliance on RNA sequencing of in vitro static culture of tail tendon fibroblasts. This aspect of the study is limited both due to concerns about the relevance of tail tendon fibroblasts to understanding tendon/enthesis pathology, and the lack of in vivo validation of these findings. The bulk of the discussion is focused on these findings rather than on the very striking in vivo findings. Specific comments below.

SUGGESTIONS TO AUTHORS

Major Comments
1. The relevance of tail tendon fibroblasts to understand Achilles tendon/ enthesis pathology is unclear, and the limitations of this approach should be addressed. Moreover, the discussion is heavily reliant on these findings, and there is insufficient in vivo validation of these findings, as well as lack of validation of the spatial transcriptomic data.
2. Disrupted fusion at 8 weeks- OCT- this is somewhat difficult to appreciate in the OCT images provided- it would be helpful to provide dissection images, or these claims should be scaled back.
3. Is growth plate failure typical in WT animals with this testing setup? It is somewhat surprising that the midsubstance is stronger than the growth plate thought this may be because the growth plate is not fully mature at this time. This should be discussed.
4. Greater annotation of structures (e.g., tide mark) in Figure 6 would improve readability. In addition, images without the background lightened should be provided (perhaps as a supplement or with the current images as an inset) to provide full picture of staining.
5. I would suggest including the TEM data as a main figure rather than a supplement.
6. The discussion of Hif1a maintaining a fibroblast 'progenitor' phenotype (in vitro) are somewhat confusing, and perhaps this is more a semantics comment, but it is unclear what this progenitor phenotype means in the context of tendon/ tendon cell biology. Moreover, greater discussion of the functional consequences of increased apoptosis in the conditional deletion entheses would better frame these findings.

Reviewer 2

SUMMARY OF THE ADVANCE MADE IN THIS PAPER AND ITS POTENTIAL SIGNIFICANCE TO THE FIELD

The manuscript from Steltzer et al., describes the role of Hif1a in the postnatal tendon and enthesis. The authors show that the enthesis remains hypoxic after birth and seek to examine the role of Hif1a, a transcription factor that regulates cellular response to hypoxic environments, in enthesis development. Using Scx-Cre to conditionally knock-out (cKO) Hif1a in Scx-expressing cells, the authors present a thorough characterization of the mutant phenotype ranging from tendon and bone morphology, size, function, mechanical properties, cell viability, histological, and molecular changes. The authors find that cKO of Hif1a disrupts enthesis ECM deposition, resulting in abnormal structural and mechanical properties. Using mouse tail tendon fibroblasts, they show that loss of Hif1a dampens the response to hypoxia and alters gene expression. They also use spatial sequencing, Seq-Scope, to demonstrate that the loss of Hif1a reduces gene expression differences between tendon and bone, suggesting that tendon differentiation processes were disrupted. Altogether, this work is the first to establish the effects of Hif1a loss on the enthesis, which would be of interest to the musculoskeletal community. Some questions remain about the data and interpretations that are listed below:

SUGGESTIONS TO AUTHORS

© 2026. Published by The Company of Biologists under the terms of the Creative Commons Attribution License (https://creativecommons.org/licenses/by/4.0/).

The authors state that "Additionally, cKO insertional tendons had more cells (higher cell density) compared to Ctrl tendons at P56 (Fig. 6B). This may be caused by a localized shift in tendon compression in cKO mice." This seems inconsistent with the other data showing increased TUNEL at P14 and decreased gene expression associated with proliferation in their RNA-seq analysis. It would be helpful to discuss these discrepancies.

The authors describe defects in the growth plates of their cKO mice "We also saw a severe disruption and loss of Safranin-O+ growth plates of cKO mice… (Fig. 6B)." Since the authors describe the previous studies of Hif1a in chondrocytes and the growth plate, it would be helpful to clarify for a reader about this phenotype. Specifically, it seems important to mention that this phenotype is likely a direct effect of Hif1a loss since Scx-Cre, while a tendon marker, is also thought to be active in cells in the growth plate.

The authors report changes in gene expression "In both Ctrl and cKO TFs, we found that 1% O2 led to downregulation of mechanoresponsive genes", yet in the figure the p values for the 20% to 1% changes are >0.05, which is not significant. Some of these p values are quite strange given the data points for each biological replicate appear to be quite tight and quite different from the comparison group (for example, Hilpda and Igfbp9 for the cKO 20% vs 1% values seem relatively separated). Can the authors clarify what gene changes are significant?

In the following sentence, do the authors mean osteoblasts rather than osteoclasts? Osr2 is generally an osteoblast marker (PMID: 17547533).
"We also identified enrichment of molecular pathways associated with ECM deposition and remodeling, including osteoclast differentiation (Osr2,Runx2, Smad6, Smad7, Smad9, Bmp4, Tgfbr1, Tgfbr2), proteoglycans, and regulation of the actin cytoskeleton (Fig. 7B and C, and Supplemental Fig. S5)."

Regarding the use of suppressed to describe decreased gene expression: for example, "Loxl and Col4 expression were suppressed". Is there a known mechanism of suppression/inhibition of gene expression? If this is unknown, it is suggested to use "decreased" instead of suppressed as the use of "suppressed" suggests an active mechanism of inhibition of transcription rather than a relative change in transcription, which could result from multiple mechanisms that alter transcript counts.

It is confusing if the cKOs generate significantly more matrix or not (Fig 8 and Fig S6C). The authors state that "Newly synthesized and secreted (nascent) matrix area was increased, although not significantly, in cKO cells cultured in normoxia compared to Ctrl cells, suggesting the cKO cells may generate more ECM under normoxic compared to hypoxic conditions (Fig. 8)." It is also stated in the discussion that they "showed that the ability of tendon progenitors to deposit ECM was also dependent on Hif1a". If the comparison of values is not significant, it seems this should not be the conclusion. They also report that hypoxia reduced GAG at 2 weeks in cKOs (Sup Fig S6B), which appears to be opposite to increased ECM.
Related to this, Fig S6C has p=0.0337 listed, but it appears that comparison was made between 1% control to 20% cKO, which does not seem to be an appropriate comparison. The same comparison between 20% Control and 1% cKO appears to be made for Fig S6B. Can the authors clarify what data was being compared regarding the generation of matrix and GAG? It seems that 20% Control should be compared to 20% cKO instead of 1% cKO.

Table 1 is present in the text, but there is no specific reference to it in the results section. How does it relate to Figure S6? Are these the same data (mean of the data) that is in Figure S6?

For Fig 9A, the cKO RNA image looks like there is a large difference in transcripts in the upper portion of the image compared with the lower portion. Is there a difference in reads per cell or regional read distribution from this difference in intensity? Since counts were normalized to the number of reads within each ROI drawn, could this have affected the analysis if a portion of one of the ROI has a higher read count than the other ROI?

For Fig 9E, the differences between bone in control and cKO compared to control tendon appear to be very different in the heatmap. It seems this would drastically affect gene expression differences that are identified when comparing bone to tendon within a sample, which seems to be the data shown in Fig 9C. Could the differences in gene expression be driven more by changes in the bone

© 2026. Published by The Company of Biologists under the terms of the Creative Commons Attribution License (https://creativecommons.org/licenses/by/4.0/).

than in the tendon? If there are drastic changes in the bone transcriptome, how can you interpret changes in the tendon between control and cKO samples? I can understand that this is a challenging experiment, but it seems that it would be informative to compare between Control and cKO tissues more directly. Alternatively, would it be possible to compare within each sample to a tissue that is not affected by the loss of Hif1a (i.e. the genes expressed in this tissue would not be expected to change between control and cKO samples)? For example, would it help to add a reference ROI to include a region or regions that are not subject to Hif1a loss by Scx-Cre and each bone or tendon region then would be compared with this non-related tissue ROI as a way to standardize between samples?

In regards to the methods of their spatial sequencing, the authors report "Differentially expressed genes were identified as genes with at least 5 reads in a sample with a p-value of 0.1 in comparisons within a section". This seems to be an especially low threshold of reads per gene per sample and a high p value. What is the sample - the ROI or the whole section? How many reads per sample or ROI were obtained? What about UMI per cell? It would help if the authors could include information on their transcriptome capturing for both samples.

First revision

Author response to reviewers' comments

Reviewer comments are in plain text

Response to reviewers are highlighted in *italics*

Changes to manuscript text are highlighted in *underline/italics*

Reviewer 1: SUMMARY OF THE ADVANCE MADE IN THIS PAPER AND ITS POTENTIAL SIGNIFICANCE TO THE FIELD

This manuscript from Steltzer et al., is focused on the role of Hif1a during post-natal development of the tendon and enthesis. Overall, the manuscript is well-written and the data largely support the conclusions. The authors are commended on the very nice qPLI data and the relatively robust characterization of Hif1a conditional knockout animals. The primary limitation of the study is the overreliance on RNA sequencing of in vitro static culture of tail tendon fibroblasts. This aspect of the study is limited both due to concerns about the relevance of tail tendon fibroblasts to understanding tendon/enthesis pathology, and the lack of in vivo validation of these findings. The bulk of the discussion is focused on these findings rather than on the very striking in vivo findings. Specific comments below.

SUGGESTIONS TO AUTHORS

Major Comments

1. The relevance of tail tendon fibroblasts to understand Achilles tendon/ enthesis pathology is unclear, and the limitations of this approach should be addressed. Moreover, the discussion is heavily reliant on these findings, and there is insufficient in vivo validation of these findings, as well as lack of validation of the spatial transcriptomic data.

Author response:

We appreciate the reviewer's comment regarding this, and the potential limitation of the work. We have included data describing differential gene expression from bulk RNA sequencing of Achilles and tail tendons of both male and female mice (n=3 biological replicates per sex, paired Achilles/tail tendons) in our response (Figure R-1). These data showed high overlap in expression between Achilles and tail tendons, with the average similarity between Achilles tendon and tail tendon DEGs at 91.2 correlation coefficient regardless of sex. We evaluated gene expression of

© 2026. Published by The Company of Biologists under the terms of the Creative Commons Attribution License (https://creativecommons.org/licenses/by/4.0/).

several tenogenic or tendon-relevant genes (e.g., Scx, Tnc, Mkx, Loxl1, Loxl2, Loxl3, Loxl4, Col1a1, Col1a2, Col3a1) based on a Biorxiv preprint from Grossemy et al. (Unravelling the distinct phenotype and mechanosensitive properties of different tendon cell populations, 2026) and showed that expression of Scx, Tnc, Loxl1-3, and Col3a1 genes do not significantly differ between matched tail and Achilles tendons from wildtype mice. We have revised the discussion to better contextualize the use of tail tendon fibroblasts and their limitations. We also removed the emphasis on the in vitro data in the discussion to focus more on the in vivo findings, and have added the following statements pertaining to the limitations of using tail tendon fibroblasts in the context of Achilles tendon biology:
"As we used cells isolated from tail tendons for tissue culture experiments, which are positional and not load-bearing like the Achilles tendon, these cells may not fully recapitulate the microenvironment or cellular responses of the load-bearing Achilles enthesis. Additional studies using cells derived from the mouse Achilles tendons or other load-bearing tendons could better validate these findings."

We have also deemphasized the spatial transcriptomic data due to the limited replication.

2. Disrupted fusion at 8 weeks- OCT- this is somewhat difficult to appreciate in the OCT images provided- it would be helpful to provide dissection images, or these claims should be scaled back.

Author response:

We appreciate the reviewer's comment on the OCT figure and have significantly revised this manuscript to add quantitative analyses and improved clarify of the figure, which now better support our claims. The inclusion of new data, which quantified tendon organization using polarization capabilityies of OCT (i.e., measurement of the slope of retardation and the frequency associated with retardation across pOCT image stacks through the tendon thickness) further support that Hif1a knockout tendons have reduced collagen organization (e.g., lower retardation frequency and smaller area under the curve calculated using 2-dimensional fast Fourier transform, implying less organization compared to age matched wildtype littermates).

3. Is growth plate failure typical in WT animals with this testing setup? It is somewhat surprising that the midsubstance is stronger than the growth plate thought this may be because the growth plate is not fully mature at this time. This should be discussed.

Author response:

In our experience, growth plate failures of the Achilles enthesis are typical in young (8wk old) mice, as the growth plate of the calcaneus is not yet fully mature. We have acknowledged this in the paper with the statement below. This premature failure is a limitation in our study as the failure is specifically in the tendon, and a different setup to clamp tendon only (by bypassing the enthesis) would directly test tendon, rather than the tendon+ enthesis (in this current study).

"To note, in using this setup for uniaxial tensile testing of the Achilles tendon, failure at the immature growth plate in 8-week old wild-type calcanei were visualized. This is a limitation in this study as the failure is not exclusively within the tendon midsubstance."

4. Greater annotation of structures (e.g., tide mark) in Figure 6 would improve readability. In addition, images without the background lightened should be provided (perhaps as a supplement or with the current images as an inset) to provide full picture of staining.

Author response:

Thank you for this comment to improve readability on Figure 6. We have included the images without the background lightened to Figure 6, and have added an annotation for the tidemark in the Silver control image.

5. I would suggest including the TEM data as a main figure rather than a supplement.

© 2026. Published by The Company of Biologists under the terms of the Creative Commons Attribution License (https://creativecommons.org/licenses/by/4.0/).

Author response:

Thank you for this suggestion. We have included the TEM data in the main text figure (now as Figure 3B).

6. The discussion of Hif1a maintaining a fibroblast 'progenitor' phenotype (in vitro) are somewhat confusing, and perhaps this is more a semantics comment, but it is unclear what this progenitor phenotype means in the context of tendon/ tendon cell biology. Moreover, greater discussion of the functional consequences of increased apoptosis in the conditional deletion entheses would better frame these findings.

Author response:

We attempted to address this concern by deemphasizing the progenitor and differentiation conclusions drawn based on in vitro and spatial transcriptomic data. We have identified the cells we cultured as fibroblasts derived from adult mouse tail tendon and have added discussion of the consequences of increased apoptosis in the conditional deletion entheses.

Revised statement in the discussion section:

"The structural deficiencies we found in the enthesis and tendon are associated to underlying changes in TF biology. TFs are the primary architects of the collagen-rich tendon ECM, which is crucial for mechanical strength.[26] During fetal development, tendon resident cells are highly proliferative, then shift to robust ECM production during postnatal growth.[27] In other mesenchymal populations, hypoxia induces a switch from oxidative phosphorylation and mitochondrial respiration to glycolysis, a pathway essential for cell survival under low oxygen conditions.[36] Chondrocyte survival in growth plate hypoxia, for instance, relies on the suppression of mitochondrial respiration.[17,37,38] We characterized the metabolic shifts typically associated with hypoxia in cells derived from tendon. Our transcriptomic profiling of TFs revealed that Hif1a-deficient cells were less capable of executing these metabolic switches, supporting the idea that hypoxic adaptation in the tendon requires HIF-1a. Interestingly, despite classical concepts, we did not observe increased TF proliferation or enhanced ECM deposition in vitro under hypoxic conditions, suggesting that tendon fibroblast responses to hypoxia may be more nuanced, and perhaps dependent on additional factors. Further, our spatial transcriptomic data unveiled a possible dysregulation of cell proliferation and growth pathways at the enthesis in which the cells may be signaled to proliferate but are failing during the cell cycle due to DNA damage, possibly leading to the dysregulation of cell population homeostasis. In tandem with increased TUNEL staining in the developing cKO enthesis, these transcriptional findings indicate that knockdown of Hif1a in tendon-resident cells disrupts cell-cycle and apoptosis pathways, likely contributing to the structural and mechanical defects observed during tendon and enthesis development."

Reviewer 2: SUMMARY OF THE ADVANCE MADE IN THIS PAPER AND ITS POTENTIAL SIGNIFICANCE TO THE FIELD

The manuscript from Steltzer et al., describes the role of Hif1a in the postnatal tendon and enthesis. The authors show that the enthesis remains hypoxic after birth and seek to examine the role of Hif1a, a transcription factor that regulates cellular response to hypoxic environments, in enthesis development. Using Scx-Cre to conditionally knock-out (cKO) Hif1a in Scx- expressing cells, the authors present a thorough characterization of the mutant phenotype ranging from tendon and bone morphology, size, function, mechanical properties, cell viability, histological, and molecular changes. The authors find that cKO of Hif1a disrupts enthesis ECM deposition, resulting in abnormal structural and mechanical properties. Using mouse tail tendon fibroblasts, they show that loss of Hif1a dampens the response to hypoxia and alters gene expression. They also use spatial sequencing, Seq-Scope, to demonstrate that the loss of Hif1a reduces gene expression differences between tendon and bone, suggesting that tendon differentiation processes were disrupted. Altogether, this work is the first to establish the effects of Hif1a loss on the enthesis, which would be of interest to the musculoskeletal community.

© 2026. Published by The Company of Biologists under the terms of the Creative Commons Attribution License (https://creativecommons.org/licenses/by/4.0/).

Some questions remain about the data and interpretations that are listed below:

SUGGESTIONS TO AUTHORS

1. The authors state that "Additionally, cKO insertional tendons had more cells (higher cell density) compared to Ctrl tendons at P56 (Fig. 6B). This may be caused by a localized shift in tendon compression in cKO mice." This seems inconsistent with the other data showing increased TUNEL at P14 and decreased gene expression associated with proliferation in their RNA-seq analysis. It would be helpful to discuss these discrepancies.

Author response:

We appreciate the reviewer's comments highlighting discrepancies between increased TUNEL and decreased proliferation gene expression. The cKO insertional tendons had an increased cell density compared to the Ctrl and increased apoptotic (TUNEL) staining. Additionally, the bulk RNA-seq data from in vitro studies showed "cKO TFs had upregulated expression of genes associated with cell cycle, proliferation, and survival…" and the spatial transcriptomic data revealed downregulation of genes associated with proliferation, and regulation of cell cycle and cell growth. To address these discrepancies, we've added the following statement in our discussion section:

"In tandem with increased TUNEL staining in the developing cKO enthesis, these transcriptional findings indicate that knockdown of Hif1a in tendon-resident cells disrupts cell-cycle and apoptosis pathways, likely contributing to the structural and mechanical defects observed during tendon and enthesis development. Notably, these signals are likely spatially and temporally heterogeneous as apoptosis appears enriched in the developing enthesis, whereas the increased cell density observed later in the insertional tendon. This may reflect compensatory or maladaptive remodeling, consistent with an overall dysregulation or imbalance of proliferation and apoptosis."

2. The authors describe defects in the growth plates of their cKO mice "We also saw a severe disruption and loss of Safranin-O+ growth plates of cKO mice… (Fig. 6B)." Since the authors describe the previous studies of Hif1a in chondrocytes and the growth plate, it would be helpful to clarify for a reader about this phenotype. Specifically, it seems important to mention that this phenotype is likely a direct effect of Hif1a loss since Scx-Cre, while a tendon marker, is also thought to be active in cells in the growth plate.

Author response:

Thank you for this comment regarding the targeted Hif1a constitutive knockdown using ScxCre. We have provided an additional paragraph (see below) in our discussion addressing this concern.

"While our study focuses on tendon-resident Hif1a function using constitutive ScxCre, it is important to recognize that Hif1a loss in broader limb mesenchymal populations can produce primary defects in endochondral skeletal development. In particular, Prrx1Cre-mediated ablation of Hif1a (in the limb bud mesenchyme) causes marked growth plate abnormalities and impaired chondrogenesis, underscoring the requirement for HIF-1A during cartilage and growth plate maturation in the embryonic limb.[11] This prior work is relevant to interpretation of ScxCre models because constitutive ScxCre does not perfectly restrict recombination to tendon fibroblasts; Scx expression is developmentally dynamic and is expressed in the skeletal primordia[37] and condensing cartilage[38], and recombination in non-tendon cells[39] likely contributes to the phenotype we observed. Consequently, some components of the tendon and enthesis phenotype could reflect a combination of tendon and enthesis-intrinsic effects of Hif1a loss and secondary consequences of altered growth plate maturation. This is particularly relevant because disruptions in growth plate development can alter long-bone growth, joint and epiphyseal geometry, and local mechanical loading, which in turn may influence enthesis loading and mineralization trajectories during postnatal maturation. In the future, more targeted gene knockdown using inducible, tendon-specific Cre lines will be worth pursuing."

© 2026. Published by The Company of Biologists under the terms of the Creative Commons Attribution License (https://creativecommons.org/licenses/by/4.0/).

3. The authors report changes in gene expression "In both Ctrl and cKO TFs, we found that 1% O2 led to downregulation of mechanoresponsive genes", yet in the figure the p values for the 20% to 1% changes are >0.05, which is not significant. Some of these p values are quite strange given the data points for each biological replicate appear to be quite tight and quite different from the comparison group (for example, Hilpda and Igfbp9 for the cKO 20% vs 1% values seem relatively separated). Can the authors clarify what gene changes are significant?

Author response:

We appreciate this comment regarding our qPCR data. We have revised the results section to reflect the significant differentially expressed genes and included only p-values less than 0.05 for clarity. We have also deemphasized the mechanoresponsive genes upon revision to the figure. Please see the statement below reflecting these changes.

"We found that mouse tail TFs from Hif1a cKO mice had reduced sensitivity to hypoxia after 1-week in culture, as indicated by damped expression of a known hypoxia-sensitive gene Hypoxia-inducible lipid droplet-associated (Hilpda or HIG-2; Fig. 7A). In Ctrl TFs, we found that 1% O2 led to downregulation of the tenogenic transcription factor, Scleraxis (Scx). A known gene target of Hif1a, Insulin-like growth factor binding protein 9 (Igfbp9) which is a member of the CCN secreted extracellular matrix associate signaling protein family, also trended down in expression in 1%O2 in both Ctrl and cKO TFs, and cKO TFs in 1% O2 had reduced expression of Scx, although not significant (Fig. 7A)."

4. In the following sentence, do the authors mean osteoblasts rather than osteoclasts? Osr2 is generally an osteoblast marker (PMID: 17547533).

"We also identified enrichment of molecular pathways associated with ECM deposition and remodeling, including osteoblast differentiation (Osr2, Runx2, Smad6, Smad7, Smad9, Bmp4, Tgfbr1, Tgfbr2), proteoglycans, and regulation of the actin cytoskeleton (Fig. 7B and C, and Supplemental Fig. S5)."

Author response:

Thank you for catching this mistake. We do mean osteoblast here and have corrected this accordingly.

5. Regarding the use of suppressed to describe decreased gene expression: for example, "Loxl and Col4 expression were suppressed". Is there a known mechanism of suppression/inhibition of gene expression? If this is unknown, it is suggested to use "decreased" instead of suppressed as the use of "suppressed" suggests an active mechanism of inhibition of transcription rather than a relative change in transcription, which could result from multiple mechanisms that alter transcript counts.

Author response:

Thank you for this suggestion. We agree that decreased gene expression is a better way to describe our results here as we do not know whether there is an active mechanism of inhibition of transcription at play here. Please see the statement below with the change incorporated.

"We also found that, in cKO TFs, the expression of Loxl and Col4 expression were reduced, as was Myh9/10, suggesting a potential role for Hif1a in ECM crosslinking and cytoskeletal organization (Fig. 7D). Bmp4 was upregulated in cKO TFs compared to Ctrl TFs, while the expression of Notch, Smad, Runx2, and Id1 were reduced (Fig. 7D)."

6. It is confusing if the cKOs generate significantly more matrix or not (Fig 8 and Fig S6C). The authors state that "Newly synthesized and secreted (nascent) matrix area was increased, although not significantly, in cKO cells cultured in normoxia compared to Ctrl cells, suggesting the cKO cells may generate more ECM under normoxic compared to hypoxic conditions (Fig. 8)." It is also stated in the discussion that they "showed that the ability of tendon progenitors to

© 2026. Published by The Company of Biologists under the terms of the Creative Commons Attribution License (https://creativecommons.org/licenses/by/4.0/).

deposit ECM was also dependent on Hif1a". If the comparison of values is not significant, it seems this should not be the conclusion. They also report that hypoxia reduced GAG at 2 weeks in cKOs (Sup Fig S6B), which appears to be opposite to increased ECM.

Related to this, Fig S6C has p=0.0337 listed, but it appears that comparison was made between 1% control to 20% cKO, which does not seem to be an appropriate comparison. The same comparison between 20% Control and 1% cKO appears to be made for Fig S6B. Can the authors clarify what data was being compared regarding the generation of matrix and GAG? It seems that 20% Control should be compared to 20% cKO instead of 1% cKO.

Author response:

We appreciate the request for clarification from this reviewer. We have revised Figure 8 and S6 to only include p-values <0.05 for clarity. We have also revised the text describing the results from this figure to reflect only the significant changes, and explicitly mentioned what may be trending although not significant. Further, we revised the text to focus on the appropriate comparisons (e.g., 20% Ctrl to 1% Ctrl, 20% cKO to 1% cKO, 20% Ctrl to 20% cKO and 1% Ctrl to 1% cKO) Please see the revised statement below. Upon reviewing the data, we agree with the reviewers' findings that it is not appropriate to conclude that the ability of tendon progenitors to deposit ECM was dependent on Hif1a, but that it may be dependent on Hif1a as the data shows trends, although not significant. Additionally, our GAG data showed a more oxygen-dependent mechanism at play as opposed to a Hif1a-dependent mechanism.

"Lastly, hypoxic conditions increased calcium and reduced ATP availability, while GAG production trended down in hypoxia at 2-weeks regardless of genotype (Supplemental Fig. S6B)."

"We showed that the ability of TFs to deposit ECM may be dependent on Hif1a, and we have identified potential regulators of cell function including cell cycle and ECM regulation that were independent of hypoxia."

7. Table 1 is present in the text, but there is no specific reference to it in the results section. How does it relate to Figure S6? Are these the same data (mean of the data) that is in Figure S6?

Author response:

Thank you for this comment. The data in this table is already in Figure 8 and S6 and we have decided to remove the table as it does not provide any additional information.

8. For Fig 9A, the cKO RNA image looks like there is a large difference in transcripts in the upper portion of the image compared with the lower portion. Is there a difference in reads per cell or regional read distribution from this difference in intensity? Since counts were normalized to the number of reads within each ROI drawn, could this have affected the analysis if a portion of one of the ROI has a higher read count than the other ROI?

Author response:

We appreciate the reviewer's careful observation. We agree that the brighter upper portion of the original cKO tendon ROI raised the possibility of uneven local read depth within the selected region, which could bias comparisons when counts are normalized to the number of reads per ROI. To address this directly, we quantified total reads per ROI in the originally submitted analysis and found substantial differences across ROIs, particularly in the cKO tendon ROI: Original ROIs (reads/ROI): Ctrl bone 32,006; Ctrl tendon 34,661; cKO bone 52,655; cKO tendon 142,477. Because the cKO tendon ROI had markedly higher total reads and included the visually brighter region, we repeated the analysis after redefining ROIs to (i) more closely match ROI areas across tissues (e.g., Ctrl bone vs Ctrl tendon) and (ii) exclude the bright region in the cKO tendon where read depth may differ (see revised spatial transcriptomic figure). Revised ROIs (reads/ROI): Ctrl bone 15,054; Ctrl tendon 21,368; cKO bone 13,287; cKO tendon 38,494. Genes detected per ROI: Ctrl bone 5,032; Ctrl tendon 5,830; cKO bone 4,778; cKO tendon 7,475. Importantly, after ROI redefinition and reanalysis, the principal pathway-level conclusions were

© 2026. Published by The Company of Biologists under the terms of the Creative Commons Attribution License (https://creativecommons.org/licenses/by/4.0/).

consistent with the original submission: genes associated with HIF-1 signaling/metabolic pathways remained among the differentially expressed genes, including Aldoa and Ldha (consistent with anaerobic metabolic pathways downstream of Hif1a signaling; notably enriched in bone). Thus, while we acknowledge that normalization to total reads per ROI can influence relative expression and that local heterogeneity in read depth is a valid concern, our revised ROI selection reduces that bias and yields qualitatively similar results. We have updated the figure and text to reflect this reanalysis and have added the read-depth information for transparency. To address this comment, we have updated Fig 9A with revised ROIs, added ROI read counts and genes detected per ROI to the methods/results, and revised the results/discussion language to reflect the exploratory nature and limitations.

9. For Fig 9E, the differences between bone in control and cKO compared to control tendon appear to be very different in the heatmap. It seems this would drastically affect gene expression differences that are identified when comparing bone to tendon within a sample, which seems to be the data shown in Fig 9C. Could the differences in gene expression be driven more by changes in the bone than in the tendon? If there are drastic changes in the bone transcriptome, how can you interpret changes in the tendon between control and cKO samples? I can understand that this is a challenging experiment, but it seems that it would be informative to compare between Control and cKO tissues more directly. Alternatively, would it be possible to compare within each sample to a tissue that is not affected by the loss of Hif1a (i.e. the genes expressed in this tissue would not be expected to change between control and cKO samples)? For example, would it help to add a reference ROI to include a region or regions that are not subject to Hif1a loss by Scx-Cre and each bone or tendon region then would be compared with this non-related tissue ROI as a way to standardize between samples?

Author response:

We agree with the reviewer that the heatmap patterns suggest that bone and tendon differ substantially and that genotype-associated changes may be driven more strongly in one tissue compartment (particularly bone). This is a key limitation of the current spatial dataset: comparisons performed within a section (e.g., tendon vs bone) can indeed yield differential expression patterns that reflect changes in either tissue, making it difficult to attribute differences specifically to tendon (or to separate tendon-intrinsic effects from altered bone state).

In response, we took several steps:

1. *Revised ROI definition and reanalysis (described in Response #1) to minimize technical sources of variability (ROI size differences; inclusion of bright/high-read-depth regions). The updated read depth distribution is substantially less extreme than the original and the revised analysis supports similar pathway-level findings.*
2. *Reframed interpretation in the text. We now explicitly state that this experiment is exploratory and that it is not powered nor structured to make strong genotype-specific conclusions, particularly regarding tendon-specific effects independent of bone changes.* We have revised the Results and Discussion to avoid over-interpretation.

Regarding the reviewer's suggestion of direct control vs cKO tissue comparisons and/or including an unaffected reference ROI: we agree this would strengthen interpretability. In the current dataset, however, we cannot guarantee the presence of a truly genotype-unaffected "reference" tissue within the same section that is also technically comparable (capture efficiency, cellular composition, and spatial context). Because of these constraints, we have not used a reference ROI normalization strategy in this revision. Instead, we now clearly acknowledge that genotype comparisons are limited in this platform/experimental design and state that future bulk RNA-seq or alternative spatial transcriptomics approaches (with increased replication and/or built-in normalization strategies) will be required to robustly define genotype- specific tendon and bone changes. We have revised the results/discussion to clarify that tendon-specific conclusions (control vs cKO) cannot be made definitively from the current spatial dataset. We also added language noting that future approaches (bulk RNA-seq and/or higher-replicate spatial methods) are needed for genotype-specific inference.

10. In regards to the methods of their spatial sequencing, the authors report "Differentially

© 2026. Published by The Company of Biologists under the terms of the Creative Commons Attribution License (https://creativecommons.org/licenses/by/4.0/).

expressed genes were identified as genes with at least 5 reads in a sample with a p-value of 0.1 in comparisons within a section". This seems to be an especially low threshold of reads per gene per sample and a high p value. What is the sample - the ROI or the whole section? How many reads per sample or ROI were obtained? What about UMI per cell? It would help if the authors could include information on their transcriptome capturing for both samples.

Author response:

We thank the reviewer for raising this important methodological point. In our original submission, "sample" referred to the ROI (not the entire section). We agree that the stated criteria (≥5 reads; p=0.1) were permissive and that readers need clearer reporting of sequencing depth/capture metrics per ROI.
To address this, we have:

1. *Clarified terminology in the Methods to explicitly state that differential expression testing was performed between ROIs within a section (ROI as the unit).*
2. *Added ROI sequencing depth and detection metrics to the manuscript. Specifically:*
   - *Reads/ROI: Ctrl bone 15,054; Ctrl tendon 21,368; cKO bone 13,287; cKO tendon 38,494.*
   - *Genes detected per ROI: Ctrl bone 5,032; Ctrl tendon 5,830; cKO bone 4,778; cKO tendon 7,475.*
3. *Raised the read-count threshold used for gene inclusion. We previously used a 5-read cutoff prior to differential testing. Based on reviewer feedback and re-evaluation, we updated this to a more stringent 10-read cutoff and repeated the analyses. The revised analysis produced qualitatively similar pathway-level results (e.g., persistence of HIF/metabolic-related genes among DEGs), supporting that the main observations were* not dependent on the lower cutoff.

Regarding UMI per cell: our spatial profiling is ROI-based rather than single-cell resolved; thus UMI-per-cell metrics are not applicable in the same way as single-cell RNA-seq. We have instead reported ROI-level read depth and genes detected, which more directly reflect capture performance for this platform and analysis unit.

Figures included to address reviewers' comments:

NOTE: We have removed unpublished data that had been provided for the referees in confidence.

Figure R-1: Bulk RNA sequencing of native mouse tail tendon and Achilles tendon revealed top variable genes were sex-dependent while most tenogenic genes did not vary greatly between tail tendon and Achilles tendon. Tail tendon and Achilles tendon were isolated from six, 6-month old C57BL6/J mice (N = 3 male and 3 female) using a Trizol-based cold pulverization RNA extraction method with an Invitrogen PureLink RNA Kit (Fisher Scientific). RNA was quantified and sequenced for up to 10M deduplicated Illumina reads using a commercial vendor (Plasmidsaurus). Bulk RNA-sequencing of 3 male and 3 female Achilles tendon and tail tendon samples clustered first on tissue type (e.g., Achilles tendon vs. tail tendon), then on sex. (A) Heatmap of top 30 genes and (B) PCA clustering of samples. (C) Volcano plot of genes upregulated in Achilles tendon (blue) versus tail tendon (red). Green indicates top 30 most variable genes. (D) Heatmap of tendon and ECM genes of interest (DEGs labeled in volcano plot). (E) Functional enrichment analyses displaying top pathways enriched containing DEGs corresponding to volcano blot. Of the top 30 genes, 3 were Y-chromosome linked while the rest clustered largely based on tissue type. Fold change ≥ 2; Adjusted p-value ≤ 0.05. AT = Achilles Tendon; TT = Tail Tendon; M = Male; F = Female.

Revised Figure 6 to improve readability:

© 2026. Published by The Company of Biologists under the terms of the Creative Commons Attribution License (https://creativecommons.org/licenses/by/4.0/).

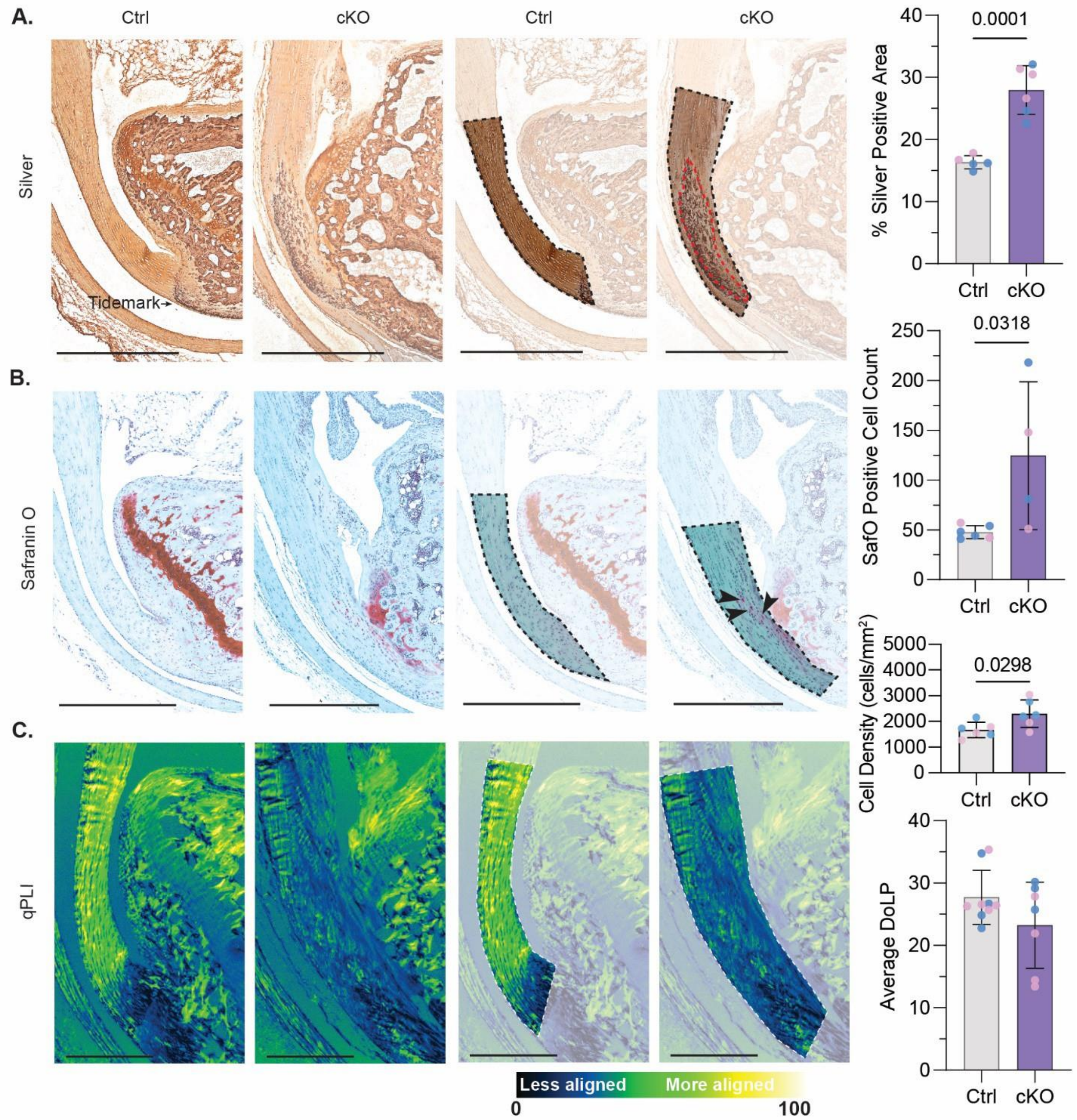

Revised Figure 3 to include TEM in main figure and include additional OCT measurements:

© 2026. Published by The Company of Biologists under the terms of the Creative Commons Attribution License (https://creativecommons.org/licenses/by/4.0/).

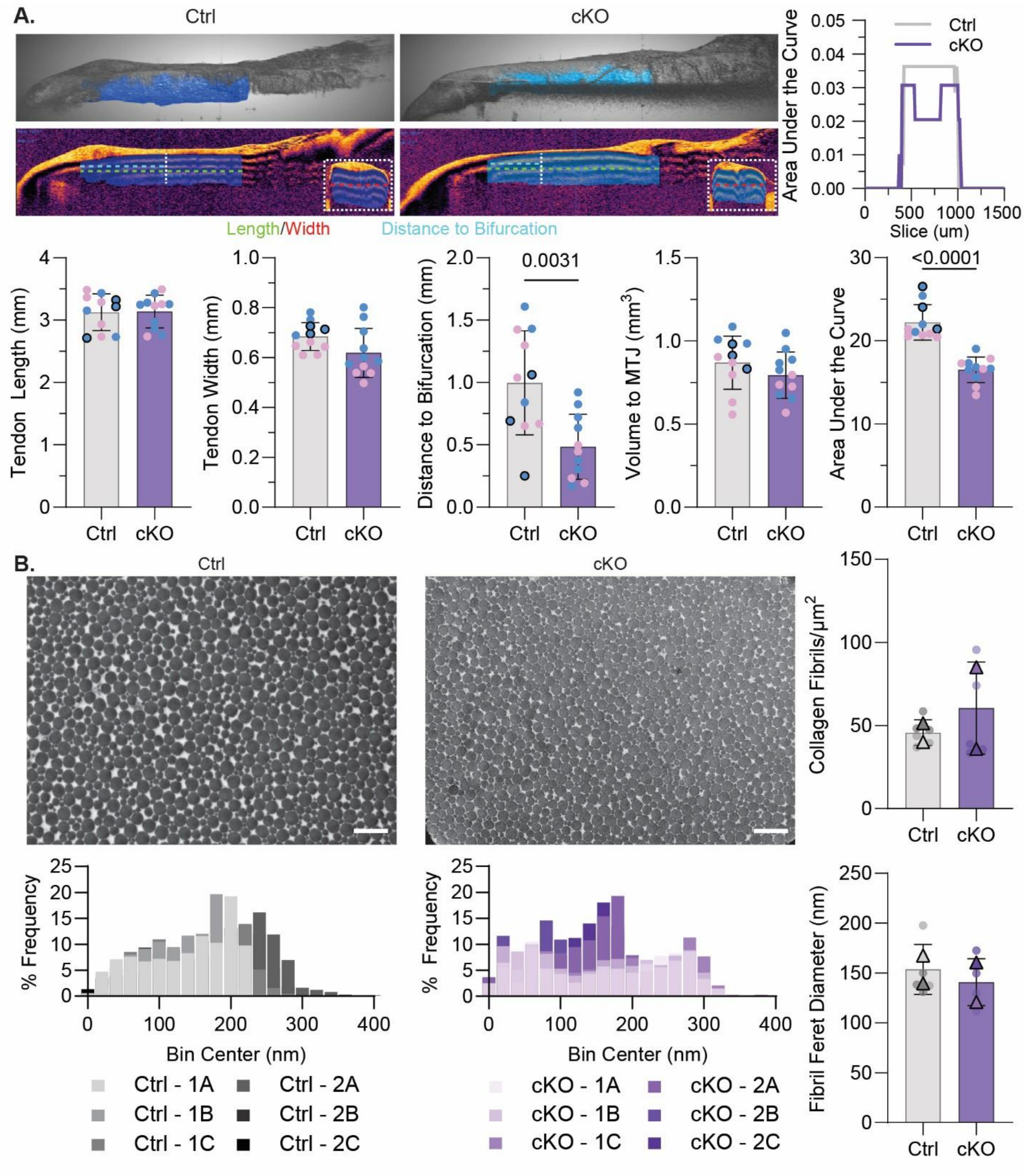

© 2026. Published by The Company of Biologists under the terms of the Creative Commons Attribution License (https://creativecommons.org/licenses/by/4.0/).

Revised Figure 8 to remove non-significant p-values:

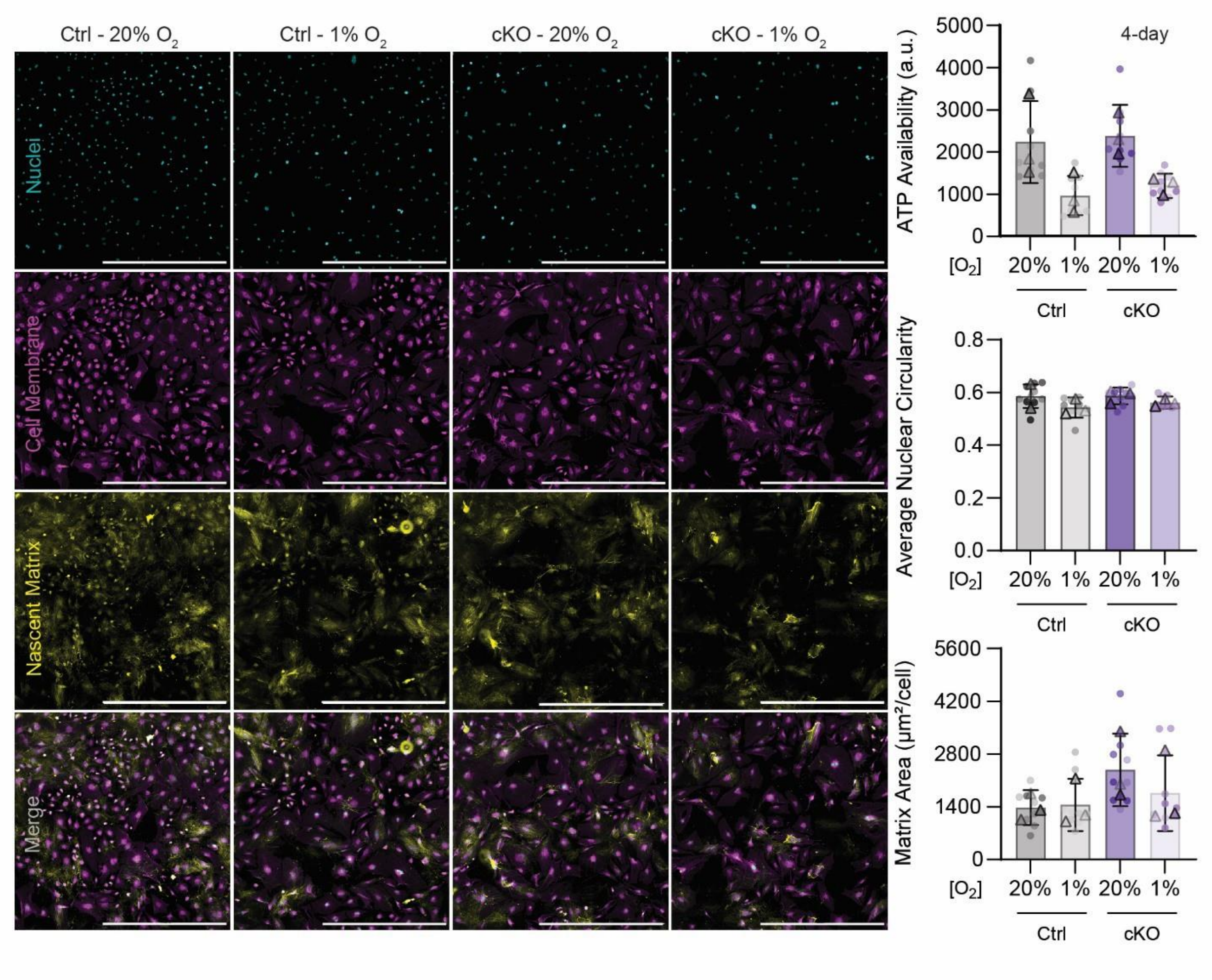

Revised Figure S6 to remove non-significant p-values:

© 2026. Published by The Company of Biologists under the terms of the Creative Commons Attribution License (https://creativecommons.org/licenses/by/4.0/).

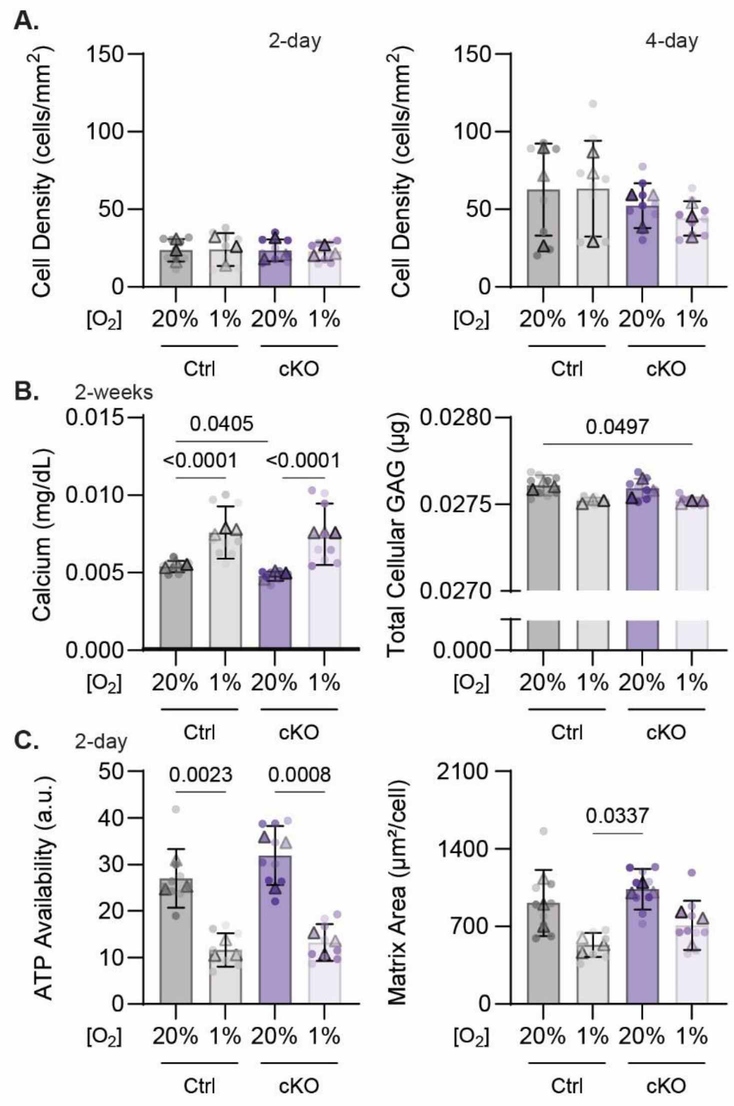

Revised Figure 9, which refined regions for analyses of tendon and simplified interpretation of results

© 2026. Published by The Company of Biologists under the terms of the Creative Commons Attribution License (https://creativecommons.org/licenses/by/4.0/).

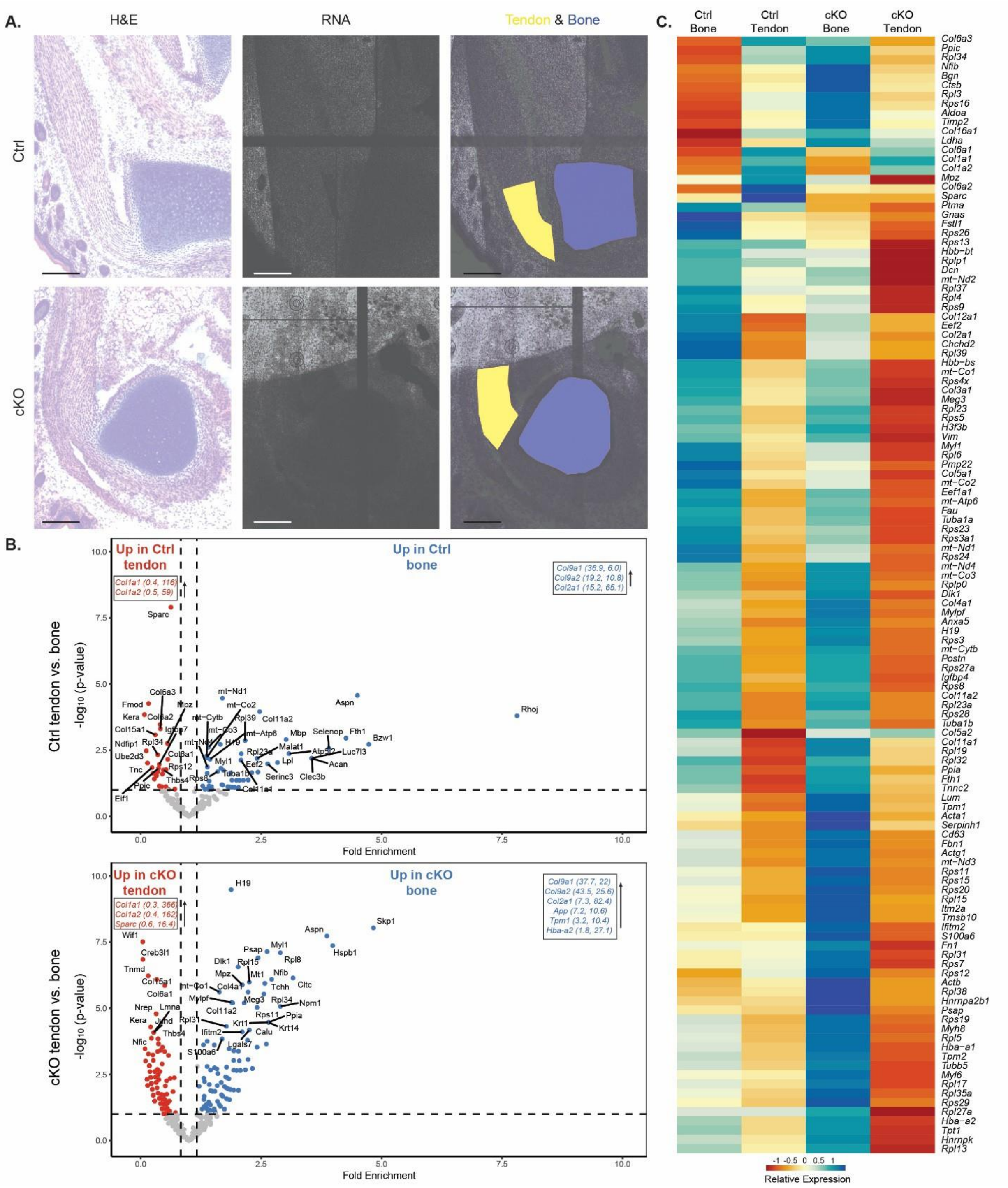

Revised Figure 2, which now includes representative biomechanics graph in panel E:

© 2026. Published by The Company of Biologists under the terms of the Creative Commons Attribution License (https://creativecommons.org/licenses/by/4.0/).

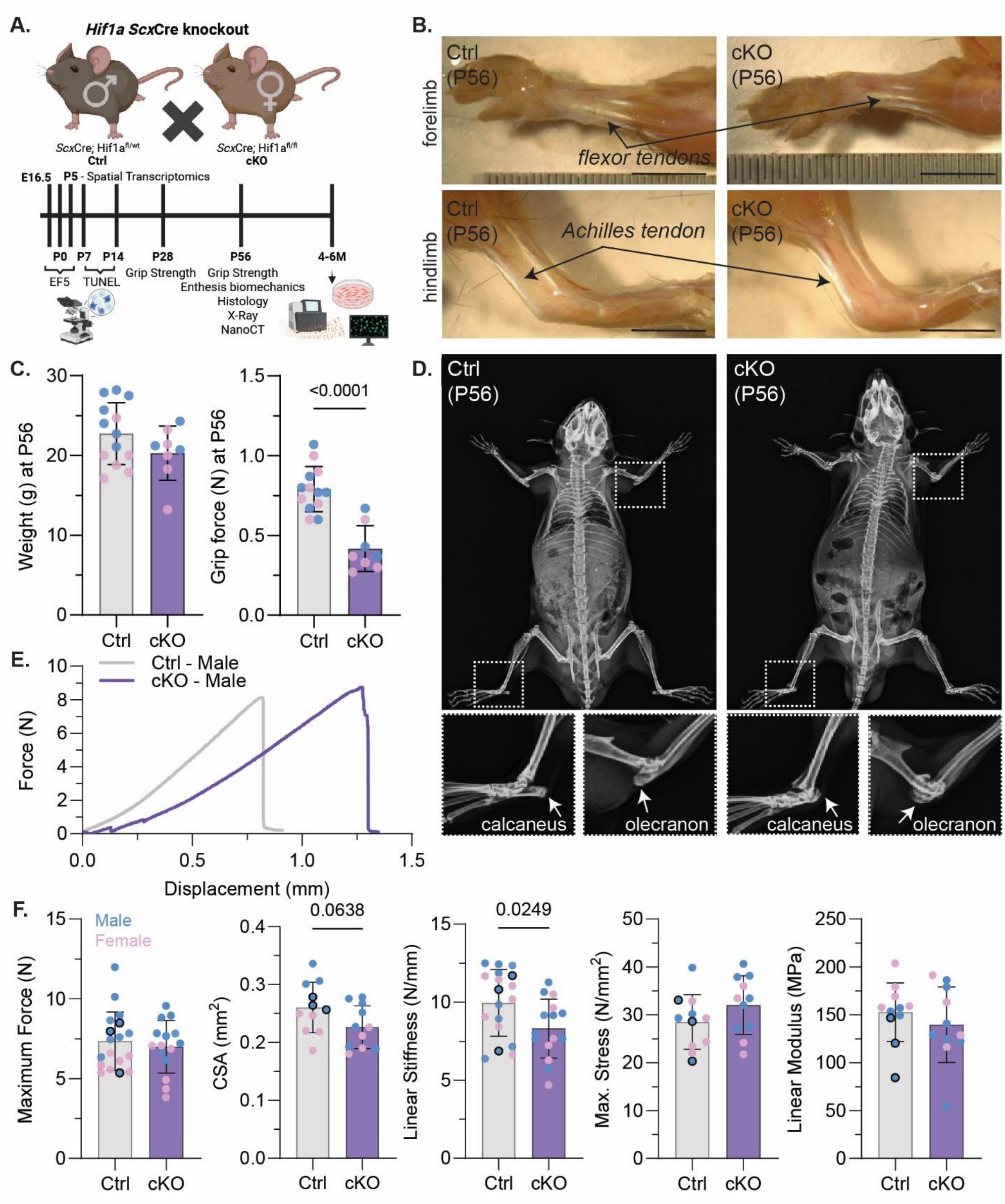

Second decision letter

MS ID#: dev.205458R1

MS TITLE: The developing tendon and enthesis are hypoxic and rely on hypoxia-inducible factor 1a (Hif1a) during postnatal development

© 2026. Published by The Company of Biologists under the terms of the Creative Commons Attribution License (https://creativecommons.org/licenses/by/4.0/).

AUTHORS: Stephanie S. Steltzer, Nicole Migotsky, Tessa Phillips, Syeda N. Lamia, Bachir A. Abeid, Kiwon Lee, Sueng-Ho Bae, Connor Leek, Sydney Grossman, Moaid Shaik, Allison Risha, Kaitlyn Frey, Claudia Loebel, Jun Hee Lee, Yatrik Shah, Adam C. Abraham and Megan Leigh Killian

Dear Dr Killian,

I am happy to tell you that your manuscript has been accepted for publication in Development, pending our standard publication integrity checks.

Reviewer 1

The authors have addressed all prior comments.

Reviewer 2

SUMMARY OF THE ADVANCE MADE IN THIS PAPER AND ITS POTENTIAL SIGNIFICANCE TO THE FIELD

This work defines the role of HIF-1a in the development of the enthesis structure. It provides a comprehensive analysis of the functional, biomechanical, structural, cellular, and molecular changes caused by the loss of HIF-1a on the tendon-bone unit. It is the first to show how the HIF-1a-mediated cell response to hypoxia is important for regulating the organization and differentiation of the enthesis.

SUGGESTIONS TO AUTHORS

The authors have addressed all of my comments.

© 2026. Published by The Company of Biologists under the terms of the Creative Commons Attribution License (https://creativecommons.org/licenses/by/4.0/).
